# A Many-Objective Optimization Algorithm Based on Weight Vector Adjustment

**DOI:** 10.1155/2018/4527968

**Published:** 2018-10-22

**Authors:** Yanjiao Wang, Xiaonan Sun

**Affiliations:** School of Electrical Engineering, Northeast Electric Power University, Jilin 132000, Jilin, China

## Abstract

In order to improve the convergence and distribution of a many-objective evolutionary algorithm, this paper proposes an improved NSGA-III algorithm based on weight vector adjustment (called NSGA-III-WA). First, an adaptive weight vector adjustment strategy is proposed to decompose the objective space into several subspaces. According to different subspace densities, the weight vector is sparse or densely adjusted to ensure the uniformity of the weight vector distribution on the Pareto front surface. Secondly, the evolutionary model that combines the new differential evolution strategy and genetic evolution strategy is proposed to generate new individuals and enhance the exploration ability of the weight vector in each subspace. The proposed algorithm is tested on the optimization problem of 3–15 objectives on the DTLZ standard test set and WFG test instances, and it is compared with the five algorithms with better effect. In this paper, the Whitney–Wilcoxon rank-sum test is used to test the significance of the algorithm. The experimental results show that NSGA-III-WA has a good effect in terms of convergence and distribution.

## 1. Introduction

Many-objective optimization problems (MAOPs) [1] refer to optimization problems whose number of objectives is over three and need to be processed simultaneously. As the number of objectives increases, the number of nondominated solutions will grow explosively in the form of *e* exponent [2], most of the solutions are nondominated, the pros and cons between the solutions become more difficult to be evaluated, and namely, many-objective evolutionary algorithms (MOEAs) have poor performance in convergence. In addition, the sensitivity of the Pareto front surface [3] will increase along with the increase of the spatial dimension, which makes it difficult to maintain the distribution among individuals.

In order to ensure the convergence and distribution of MOEAs [4], scholars have proposed the following three solutions to improve the aforementioned problems:

1. Change the dominance relationship [5], and then increase the pressure of selecting solution to improve the convergence of the algorithm. In 2005, Laumanns et al. proposed e-domination [6] to reduce the fitness value of each individual by (1 − *e*) times before the individual performs the Pareto dominance relationship comparison. In 2014, the *ϵ*-dominant mechanism proposed by Hernández-Díaz et al. [7] added an acceptable threshold to the comparison of individual fitness. In 2014, Yuan et al. proposed the *θ* domination [8] to maintain the balance between convergence and diversity in EMO. In 2015, Jingfeng et al. proposed a simple Pareto adaptive *ε*-domination differential evolution algorithm for multiobjective optimization [9]. In 2016, Yue et al. proposed a grid-based evolutionary algorithm [10] which modifies the dominance criterion to increase the convergence speed of evolutionary many-objective optimization (EMO). In 2017, Lin et al. proposed an evolutionary many-objective optimization based on alpha dominance, which provides strict Pareto stratification [11] to remove most of the blocked domination solutions. The methods above can enhance the selection pressure, but this relaxed strategy is limited to handle the situation with a little number of objectives. Moreover, it is very hard that parameters need to be adjusted for different optimization problems.
2. Method based on decomposition [12], which means to decompose the objective space into several subspaces without changing the dimension of the objective, thus transforming MAOP into single-objective subproblem or many-objective subproblems. In 2007, Zhang and Li proposed a many-objective evolutionary algorithm based on decomposition [13] (MOEA/D) for the first time whose convergence effect is significantly better than MOGLS and NSGA-II. In 2016, Yuan et al. proposed a distance-based update strategy, MOEA/DD [14], to maintain the diversity of algorithms in the evolutionary process by exploiting the vertical distance between solutions and weight vectors. In 2017, Segura and Miranda proposed a decomposition-based MOEA/D-EVSD [15] evolutionary algorithm, steady-state form, and a reference direction to guide the search. In 2017, Xiang et al. proposed the framework of VAEA [16] algorithm based on angle decomposition. The algorithm does not require the reference point, and the convergence and diversity of many-objective space are well balanced. However, the self-adjusting characteristics of the algorithms mentioned above make them fall into local optimum more easily although the convergence speed is improved. The distribution is not unsatisfactory.
3. The reference point method, this kind of algorithm decomposes the MAOPs into a group of many-objective optimization subproblems with simple frontier surfaces [17]. However, unlike the decomposition method, the subproblem is solved using the many-objective optimization method. In 2013, Wang et al. [18] proposed the PICEA-w-r algorithm whose set of weight vectors evolved along with populations; the weight vector adjusts adaptively according to its own optimal solution. In 2014, Qi et al. adopted an enhanced weight vector adjustment method in the MOEA/D-AWA algorithm [19]. In 2014, Deb and Jain proposed a nondominated sorting evolution many-objective optimization algorithm based on reference points [20] (NSGA-III), and its reference point is uniformly distributed throughout the objective space; in the same year, Liu et al. proposed the MOEA/D-M2M method; the entire PF can be divided into multiple segments and solved separately by dividing the entire objective space into multiple subspaces. Each segment corresponds to a many-objective optimal subproblem [21], which improves the distribution of solution sets. In 2016, Bi and Wang proposed an improved NSGA-III many-objective optimization algorithm [22] (NSGA-III-OSD) based on objective space decomposition. The uniformly distributed weight vector was decomposed into several subspaces through a clustering approach. The weight vectors can specify a unique subarea. A smaller objective space helps overcome the invalidity of the many-objective Pareto dominance relationship [23]; the distribution and the uniformity of the solution surface decrease because of the sparse solution of each subspace edge caused by the fixed subspace. In 2016, Cheng et al. proposed an evolutionary algorithm based on reference vector guidance for solving MAOPs [24] (RVEA). Its principle of the adaptive strategy is to adjust the weight vectors dynamically according to the objective function form. The weight vectors generated by the methods above are uniformly distributed, while the reference point on the solution surface cannot be guaranteed to be uniform, and there is also a possibility that the convergence may be lost.

In order to further improve the convergence and distribution of many-objective algorithms, based on NSGA-III, a many-objective optimization algorithm (NSGA-III-WA) based on weight vector adjustment is proposed. First, in order to enhance the exploration ability of the solution to the weight vector, an evolutionary model in which a novel differential evolution strategy and a genetic evolution strategy are integrated is used to generate new individuals. Then, in order to ensure the uniform distribution of weight vectors in the solution surface, the objective space is divided into several subspaces by clustering the objective vectors. According to the adjustment of the weight of each subspace, the spatial distribution of the objective is improved. We will carry out simulation experiments on the DTLZ standard test set [25] and WFG standard test set [26]. We compare the proposed algorithm with the five algorithms that are currently performing better on the optimization problem of 3 to 15 objectives. The GD, IGD, and HV are compared as performance indicators. The experimental results show that NSGAWA has good effect in convergence and distribution.

The rest of the paper is organized as follows.  Section 2 introduces the original algorithm.  Section 3 describes the proposed many-objective evolutionary algorithm.  Section 4 compares the similarities and differences between this algorithm and similar algorithms.  Section 5 gives the experimental parameters of each algorithm and comprehensive experiments and analysis. Finally, Section 6 summarizes the full text and points out the issues to be studied next.

## 2. NSGA-III

The NSGA-III algorithm is similar to the NSGA-II algorithm in that it selects individuals based on nondominated ordering. The difference is that the individual choice after nondominated sorting is different. The NSGA-III algorithm is introduced as follows:

First, a population A of size *N* is set up, and population *P*_*t*_ is operated by genetic operators (selection, reorganization, and variation) to obtain a population *Q*_*t*_ of the same size, and then population *P*_*t*_ and population *Q*_*t*_ are mixed to obtain a population *R*_*t*_ of 2*N*.

The population *R*_*t*_ is subjected to nondominated sorting to obtain layers of individuals with nondominated levels (*F*1, *F*2, and so on). Individuals with nondominated levels are sequentially added to the set *S*_*t*_ of the next generation of children until the size of the set *S*_*t*_ is greater than *N*. The nondominated level at this time is defined as the L layer. Pick *K* individuals from the L level so that the sum of *K* and all previous levels is equal to *N*. Prior to this, the objective value is normalized by the ideal point and the extreme point. After normalization, the ideal point of *S*_*t*_ is a zero vector, and the provided reference point is located exactly on the normalized hyperplane. The vertical distance between each individual in *S*_*t*_ and each weight vector (connect the origin to the reference point) is calculated. Each individual in *S*_*t*_ is then associated with a reference point having a minimum vertical distance.

Finally, the niche operation is used to select members from *F*1. A reference point may be associated with one or more objective vectors or there are also possibilities that none of the objective vectors is associated with a reference point. The purpose of the niche operation is to select the *K* closest reference points from the *F*1 layer into the next generation. Firstly, calculate the number of individuals associated with each reference point in the *S*_*t*_/*F*_*l*_ population and use *ρ*_*j*_ to represent the number of individuals associated with the *j*th reference point. The specific operation is as follows:

When the number of individuals associated with a reference point is zero, in other words, *ρ*_*j*_ is equal to zero, the operation next depends on whether there are individuals related to the reference point in *F*_*l*_. If one or more individuals are related to the reference vector, extract the point with the smallest distance, add it to the next generation, and set *ρ*_*j*_=*ρ*_*j*_+1. If no individual is associated with the reference point in *F*_*l*_, the reference point vector in this generation will be deleted. If *ρ*_*j*_ > 0, choose the nearest reference point until the population size is *N*.

## 3. The Proposed Algorithm

### 3.1. The Proposed Algorithm Framework

In order to further improve the convergence speed and distribution of NSGA-III algorithm, a multiobjective optimization algorithm based on weight vector adjustment (NSGA-III-WA) is proposed.  Algorithm 1 is the framework of the NSGA-III-WA algorithm. The algorithm is mainly improved in two aspects: evolution strategy and weight vector. This paper also adds the discriminating condition for enabling weight vector adjustment, which speeds up the running of the algorithm without affecting the performance of the algorithm. First, we initialize population *P*_*t*_ with population size *N* and weight vector *W*_unit. Secondly, we enter the algorithm iteration process, generate the population *Q*_*t*_ by the operating population *P*_*t*_ using the differential operator, and then obtain population *R*_*t*_ sized 2*N* using the combination of *P*_*t*_ and *Q*_*t*_. *R*_*t*_ should be updated through the environmental selection strategy. The next generation of population *P*_*t*+1_ is obtained. Lastly, adjust the weight vector and determine if the termination condition is satisfied. If so, output the current result and terminate the algorithm; otherwise, continue iterating.

### 3.2. Initialization

The initial population is randomly generated whose size is the same as the number of weight vectors in its space. This article uses Das and Dennis's systematic method [27] to set weight vectors *W*_unit={*w*^1^, *w*^2^,…, *w*^*N*^}. The total number of weight vectors is equal to *N*=*C*_*H*+*M*−1_^*M*−1^, where *H* represents the dimension of the solution vector and *M* is the number of objective functions. The initialized weight vectors (reference points) are uniformly distributed in the objective space, and each weight vector generation method is as follows: for *w*_*i*_ ≥ 0, *i*=1,…, *H*, ∑_*i*=1_^*H*^*w*_*i*_=1.

### 3.3. Evolutionary Strategy

The evolutionary strategy is essential to the convergence speed and accuracy of the solutions because it will determine the quality of new solutions to subquestions directly during evolutionary process. In order to improve the convergence speed, this paper proposes a new differential evolution strategy to replace the original strategy. The pseudocode is shown in Algorithm 2. Every individual performs the same operation as follows.

#### 3.3.1. Variation

It is mainly divided into two parts:

(a) Select three individuals *x*_*r*1_, *x*_*r*2_, *x*_*r*3_ randomly from the population. A new individual *x*_*v*_ will be obtained using (1) for parental vector variation to maintain population diversity. At the beginning of the algorithm, the mutation rate should be relatively large to make the individuals different from each other. This not only improves the search ability of the algorithm, but also prevents the individual from falling into local optimum. The mutation rate should decrease as the number of iterations increases and the solution approaches the Pareto optimal front surface (PFs) [28]. At this time, the mutation rate should decrease to accelerate the convergence of the algorithm to the optimal. This not only improves the convergence speed, but also reduces the complexity of the algorithm. Based on the above analysis of the needs of the algorithm, this paper proposes an adaptive mutated factor *F*=0.5+0.5cos(*π* × gen/gen_max). It can be seen that as the number of iterations increases, the mutation rate decreases in size. At the beginning of the algorithm, it can enhance the individual's ability to jump out of local optimum and find superior individuals. As the mutation rate is smaller, the algorithm tends to be stable. To maintain the diversity of populations, select individuals *x*_*r*1_, *x*_*r*2_ to generate a new individual (line 3 in Algorithm 2) by simulating the binary recombination operator (2) and (3). In (4), *u* is a random number between [0, 1] and *η* is a constant with the fixed value of 20.
  (1) $$\begin{array}{c} x_{v}=x_{r1}+\left(0.5+0.5\cos\left(\pi \times \frac{\text{gen}}{\text{gen\_max}}\right)\right)\left(x_{r2}-x_{r3}\right), \end{array}$$
  (2) $$\begin{array}{c} x_{c1,j}=0.5\left[\left(1+\gamma_{j}\right)x_{r1,j}+\left(1-\gamma_{2}\right)x_{r2,j}\right], \end{array}$$
  (3) $$\begin{array}{c} x_{c2,j}=0.5\left[\left(1-\gamma_{2}\right)x_{r1,j}+\left(1+\gamma_{j}\right)x_{r2,j}\right], \end{array}$$
  (4) $$\begin{array}{c} \gamma_{j}\left\{\begin{array}{cc} {\left(2u_{j}\right)}^{1/\left(\eta +1\right)} & \text{if }u_{j}\leq 0.5,\\ {\left[\frac{0.5}{\left(1-u_{j}\right)}\right]}^{1/\left(\eta +1\right)} & \text{otherwise}. \end{array}\right. \end{array}$$
(b) Select an individual through probability selection from *x*_*v*_, *x*_*c*1_, *x*_*c*2_ (which are generated in step a) to execute the crossover operation (lines 4 to 12 in Algorithm 2). The specific operation is as follows: Generate a random number between [0, 1] and compare it with *p*. If the number is smaller than *p*, execute lines 5–9 of the pseudocode in Algorithm 2; otherwise, select *x*_*v*_ to enter the crossover operation (the detailed operations of lines 5–9 in Algorithm 2 are as follows: Generate a random number between [0, 1] and compare it with 0.5. If it is smaller than 0.5, then select *x*_*c*2_ to enter the crossover operation. Otherwise, select *x*_*c*1_ to enter the crossover operation). Here, *p* is 0.5.

#### 3.3.2. Crossover

This article selects the single-point crossing method, which is located in lines 13–20 of the pseudocode in Algorithm 2. In this way, individual selectivity is enhanced. Generate a random number between [0, 1]. If the number is less than or equal to the crossover operator CR, then select a certain dimension of the individual randomly and execute the crossover operations at the selected point. A large number of experiments have confirmed that when the value of CR is 0.4, the effect is better. Then use the generated individuals and their fitness values to replace the original ones.

### 3.4. Environmental Selection

The purpose of the environmental selection operation is to select the next generation of individuals. The framework of Algorithm 3 includes the following steps: (1) Nondominated sorting is conducted on the population *R*_*t*_, and individuals with a rank of 1, 2, 3,… after nondominating sorting are added to the offspring collection *S*_*t*_ in order. (2) When the size of *S*_*t*_ is greater than or equal to *N*, note the nondominant level *F*_*l*_ at this time and determine when to terminate the operation (|*S*_*t*_|=*N*) or enter the next step (*S*_*t*_| > *N*). (3) Select individuals in *S*_*t*_/*F*_*l*_ and enter *P*_*t*+1_ until its size is *N*. The specific operation is discussed below.

#### 3.4.1. Normalize Objective

Since the magnitude of the respective objective values is different, it is necessary to normalize objective values for the sake of fairness. First, calculate the minimum value *z*_*i*_ of each dimension for every objective function. The sets of *z*_*i*_ constitute the ideal points. All individuals are then normalized according to (5), where *a*_*i*_ is the intercept of each dimension that can be calculated according to the achievement scalarizing function (ASF) shown in (6).

(5) $$\begin{array}{c} f_{i}^{n}\left(x\right)=\frac{{f^{\prime }}_{i}\left(x\right)}{a_{i}-z_{i}^{\min}}=\frac{f_{i}\left(x\right)-z_{i}^{\min}}{a_{i}-z_{i}^{\min}},\;\text{for }i=1,2,\ldots ,M, \end{array}$$

(6) $$\begin{array}{c} \text{ASF}\left(x,w\right)=\overset{M}{\underset{i=1}{\max}}\frac{\left(f_{i}\left(x\right)-z_{i}^{\min}\right)}{w_{i}},\;\text{for }x\in S_{t}. \end{array}$$

#### 3.4.2. Associate Each Member of *S*_*t*_ with a Reference Point

In order to associate each individual in *S*_*t*_ with the reference points after normalization, a reference line is defined for each reference point on the hypersurface. In the normalized objective space, the reference point with the shortest distance is considered to be related to population members.

#### 3.4.3. Compute the Niche Count of the Reference Point

Traversing every individual in the population, calculate the distances between itself and all reference points and record the number of individuals associated with each reference point using *ρ*_*j*_, which represents the number of individuals associated with the *j*th reference point.

#### 3.4.4. Niche Preservation Operation

If the number of populations associated with this reference point is zero (but there is an individual associated with the reference point vector in *F*_*l*_), then find the point with the smallest distance and extract it from *F*_*l*_ to join the selected next generation population. In the setting, the number of associated populations is increased by one. If each individual is not referenced to the reference point in the *F*_*l*_, the reference point vector is deleted. If the number of associated populations is not zero, then the nearest reference point is selected until the population size is *N*.

### 3.5. Weight Adjustment

The uniformity of the solution surface cannot be achieved when the algorithm reaches a certain stable state, although the weight vectors distribute uniformly in the space. This is because of the complexity caused by the irregular shape of the PFs of the objective functions. The distribution of weight vectors is particularly important when all individuals are indistinguishable from each other and locate on the first level of the dominance level. Therefore, in order to improve the distribution of many-objective algorithms, a weight vector adjustment strategy whose framework is shown in Algorithm 4 is proposed. The distribution of weight vectors is appropriately adjusted according to the shape of the nondominated frontier. In order to prevent the weight vector adjustment in the high-dimensional space from being concentrated to a certain objective, the *K*-means clustering method is used to divide the weight vector into different subspaces. The specific operations are described below.

**Figure 1:**
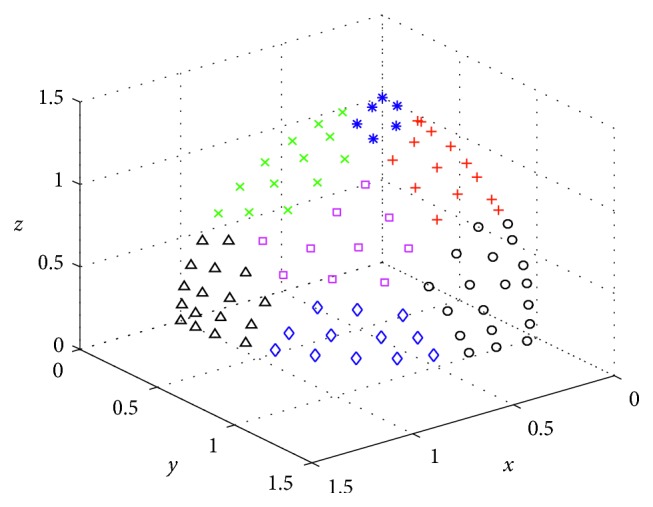
Many subspaces by using the *K*-means clustering.

**Figure 2:**
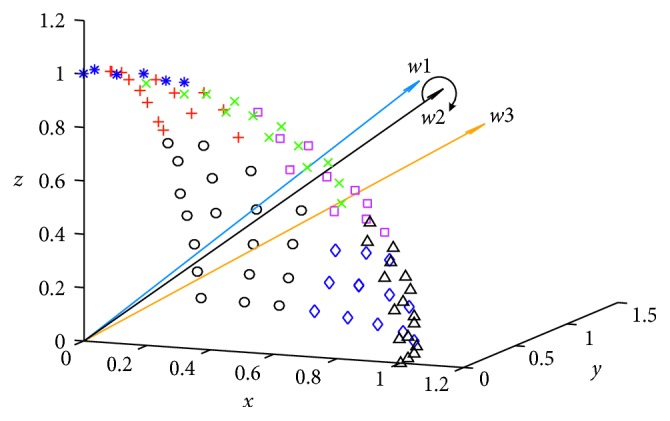
Weight adjustment.

First, each weight vector is associated with the population member (line 1 in Algorithm 4). Secondly, the solution space is decomposed into many subspaces using the *K*-means clustering method (lines 2–5 in Algorithm 4), as shown in Figure 1. To prevent errors caused by excessive differences in the solution set in space, the subspace should not be too large or small, and it can be divided into *C* spaces according to the size of the population. A large number of experiments confirmed that when *C* = 13, better results can usually be obtained. The solution set is decomposed into [*N*/*C*] cluster spaces, and the weight vectors are adjusted by comparing the density of the entire objective space and subspaces (lines 6–17 in Algorithm 4). As shown in Figure 2, *w*2 should be away from *w*1 and approach *w*3. Finally, the number of weight vectors is adjusted to ensure that it can match the original number. If the number is greater than *N*, then the weight vector is deleted at the densest position in the entire objective space. If the number is less than *N*, then a weight vector is added in sparse position (lines 18–20 in Algorithm 4).

The definition of spatial density is obtained by averaging the distances of similar individuals in the population. The minimum spatial density is defined as *h*_1_*ρ*_o_. The maximum spatial density is defined as *h*_2_*ρ*_o_. Under normal circumstances, when the value of *h*1 is 0.2 and *h*2 is 1.3, relatively good results can be obtained. Note *ρ*_o_ is the density of the overall objective space and *ρ*_*i*_ is density of the subspace. The adjustment process is divided into two situations described below:

(1) When the subspace density is less than the objective space density, determine whether the subspace density is too small. If the density of the subspace is less than the minimum space density *h*_1_*ρ*_o_, then the subspace density is considered too small. In this case, the weight vector should be evacuated. At this point, using the two nearest neighbor weight vectors and adding their sum vectors to the set of weight vectors, the parent vectors are deleted. Otherwise, the weight vector should be fine-tuned to achieve uniformity across the objective plane. At this time, according to the density difference, the nearest two weight vectors in the subspace are adjusted according to (7) and (8). Among them, the vectors *W*_unit(*k*) and *W*_unit(*l*) are the closest weight vectors. Let mt be the minimum distance. Let *ρ*_*i*_ be the density value. The vectors *W*_unit(*gwk*) and *W*_unit(*gwl*) are neighbor weights of the respective weights.
  (7) $$\begin{array}{c} W\text{\_unit}\left(k\right)=W\text{\_unit}\left(k\right)+\left(\rho_{i}-\text{mt}\right)\times W\text{\_unit}\left(gwk\right), \end{array}$$
  (8) $$\begin{array}{c} W\text{\_unit}\left(l\right)=\text{W\_unit}\left(l\right)+\left(\rho_{i}-\text{mt}\right)\times W\text{\_unit}\left(gwl\right). \end{array}$$
(2) When the subspace density is greater than the objective space density, determine whether the subspace density is too large. If the density of the subspace is greater than the maximum space density *h*_1_*ρ*_o_, then the subspace density is too large. In this case, the weight vector should be aggregated. At this point, take the two furthest neighboring weight vectors and add their sum vectors to the set of weight vectors. Otherwise, at this time, according to the density difference, adjust the weight vectors according to (9) and (10). Among them, the vectors *W*_unit(*k*) and *W*_unit(*l*) are the furthest weight vectors. Note mx is the maximum distance and *ρ*_*i*_ is the density value.
  (9) $$\begin{array}{c} W\text{\_unit}\left(k\right)=W\text{\_unit}\left(k\right)+\frac{\left(\text{mx}-\rho_{i}\right)}{2}\times W\text{\_unit}\left(l\right), \end{array}$$
  (10) $$\begin{array}{c} W\text{\_unit}\left(l\right)=W\text{\_unit}\left(l\right)+\frac{\left(\text{mx}-\rho_{i}\right)}{2}\times W\text{\_unit}\left(k\right). \end{array}$$

It is worth emphasizing that the edge vector is immovable; otherwise, the search range of the algorithm will be affected. Half of the maximum number of iterations was selected as an enabling condition for the weight vector adjustment strategy and adjust every four generations in this paper. In this time, the objective vectors have approached the PFs, so the guidance of the weight vectors is relatively accurate, and the population update is relatively stable (i.e., it is close to the PF).

## 4. Discussion

The previous section described the NSGA-III-WA algorithm in detail. In this section, we compare the similarities and differences between NSGA-III-WA, NSGA-III, and VAEA.

### 4.1. The Similarities and Differences between NSGA-III-WA and NSGA-III

- Both algorithms use Pareto dominance to select individuals.
- The evolution of the two algorithms is different. NSGA-III adopts the original genetic evolution strategy, while NSGA-III-WA adopts a new differential evolution strategy to optimize individuals. It has better effect on convergence speed and solution accuracy.
- Both algorithms have different strategies for dividing the objective space. The NSGA-III algorithm uses the original method of generating weight vectors to evenly divide the objective space. The NSGA-III-WA algorithm divides the objective space into several subspaces and adjusts the weight vectors according to the individual density of the objective space. This method can better ensure the uniformity of the weight vectors on the objective surface, thus ensuring the uniformity of the solution set.

### 4.2. The Similarities and Differences between NSGA-III-WA and VAEA

- Both algorithms use Pareto dominance to select individuals.
- Both algorithms need to normalize the population. The difference is that VAEA normalizes the population according to the ideal and lowest point of the population, while NSGA-III-WA obtains the intercept of each objective axis by calculating the ASF and then normalizes the population. The latter is more universal and more reasonable.
- Both algorithms have associated operations. VAEA does not relate to the association of reference points. It achieves the association between individuals and individuals and thus cannot guarantee individual distribution. NSGA-III-WA associates individuals with weight vectors to improve the distribution of the algorithm.

## 5. Simulation Results

In order to verify the performance of the proposed algorithm on MAOPs, this paper selects the general test function set DTLZ and WFG in the field of many-objective optimization for simulation experiments. The proposed algorithm is compared with five reliable algorithms, MOEA/D, NSGA-III, VAEA, RAEA, and MOEA/D-M2M, and representative algorithms with the objectives 3, 5, 8, 10, and 15 on the DTLZ1-6 test function and WFG1-4 test instances. Performance indicators GD [29], IGD [30], and HV [31, 32] are used for comparative analysis: first, to briefly introduce the corresponding parameter settings for each algorithm, and second, to explain the experimental results, compare, and analyze them.

### 5.1. General Experimental Settings

The number of decision variables for all test functions is *V* = *M* + *k* − 1, and *M* is the number of objective functions, *k* = 5 for DTLZ1, and *k* = 10 for DTLZ2-6. The number of decision variables for all WFG test functions is *V*=*k*+1, where the position variable is *k*=*M* − 1 and the objective dimension is *M*; the distance variable is *l*=10. The population sizes of NSGA-III and RVEA are related to the uniformly distributed weight scale and determined by the combination number of *M* and the number of *p* on each objective. The double-layer distribution method in [13] is adopted in order to tackle the problem. The specific parameter settings are given in Table 1. For fair comparison, the population size is the same as the other three algorithms. The algorithm runs independently for 30 times on each test function. The algorithm uses the maximum function evaluation (MFE) as the terminal condition for each run.

Due to objective dimensions of the solution problem, MFE is also not the same. According to [16], the specific settings are shown in Table 2. The maximum number of iterations is calculated by gen_max=MFE/*N*. The parameter settings of NSGA-III and MOEA/D are shown in Table 3. In addition, the number of MOEA/D neighbor weight *T* is 10; the RVEA penalty factor change rate *α* is 2, and the VAEA angle threshold is expressed as *δ*=(*π*/2)/(*N*+1).

### 5.2. Results and Analysis

In order to verify the performance saliency of the proposed NSGA-III-WA algorithm on many objective optimization problems, general performance evaluation indicators GD, IGD and HV were used. It is compared with five good algorithms, MOEA/D, NSGA-III, VAEA, RAEA, and MOEA/D-M2M, and representative algorithms with the objectives 3, 5, 8, 10, and 15 on the DTLZ1-6 test function and WFG1-4 test instances.

#### 5.2.1. Testing and Analysis of DTLZ Series Functions

This section shows the results and analysis of the GD, IGD, and HV performance test data of the DTLZ1-6 test function. The experimental results are shown in Tables 4–6. They are the average values and standard deviations of 30 independent running results. The best results are shown in black and bold, and the values in parentheses indicate the standard deviation; the number in square brackets is the algorithm performance ranking, which is based on the Whitney–Wilcoxon rank-sum test [33]. To investigate whether NSGA-III-WA is statistically superior to other algorithms, Wilcoxon's rank-sum test is performed at a 0.05 significance level between NSGA-III-WA and each competing algorithm on each test case. The test results are given at the end of each cell, represented by the symbols “+,” “=,” or “−,” which indicate that the NSGA-III performance is better than the algorithm in the corresponding column, equal to, and worse. At the same time, the last row of Tables 4–6 summarizes the number of test instances that NSGA-III-WA is significantly better than, equal to, and below its competitors. Tables 7–9 show the results of the comparison of NSGA-III-WA algorithm with the other five algorithms under different objective numbers.

It can be seen from the experimental results in Table 4 that the GD values of NSGA-III-WA on the DTLZ1-4 are superior to the other five algorithms, only 8th, 10th, and 15th dimensions are superior on the DTLZ5, and NSGA-III on the DTLZ6 gets the best results. It shows that in solving many-objective problems, the convergence of NSGA-III-WA is more effective than that of NSGA-III algorithm and is better than other algorithms.  Table 7 shows summary of statistical test results from Table 4. NSGA-III-WA is compared to five other more advanced multiobjective algorithms and counts the number of wins (+), equal to (=), and number of loses (−). As can be seen from the table, NSGA-III-WA is clearly superior to the five most advanced designs selected.

From Table 5, the NSGA-III-WA can get the best results especially the objectives 5, 10, and 15 on the DTLZ1, 8, 10, and 15 on the DTLZ2 and DTLZ3, 3, 5, and 8 on the DTLZ4, and 5, 8, and 15 on the DTLZ5. Moreover, the objectives 3 and 8 on the DTLZ1, the objectives 3 and 5 on the DTLZ2, the objective 3 on the DTLZ3, and the objective 10 on the DTLZ5 achieve the second best results. Nevertheless, on the DTLZ5 and DTLZ6, the results of NSGA-III-WA are not significant because the DTLZ5 and DTLZ6 are used to test the ability to converge to a curve. Owing to the reason that NSGA-III-WA needs to build a hypersurface, *M* extreme points cannot be found in the later stage of the algorithm to construct the hypersurface, and it cannot converge to a curve well. In addition, NSGA-III-WA has noticeable effects on other test functions and is a kind of stable and relatively comprehensive algorithm.  Table 8 shows summary of statistical test results from Table 5. It can be seen from the table that NSGA-III-WA performs best on the other five algorithms.

From Table 6, it can be seen that the NSGA-III-WA can effectively handle most test problems. It can get the best results especially the objectives 3, 8, 10, and 15 on the DTLZ1, 5 and 10 on the DTLZ2, 3, 5, 10, and 15 on the DTLZ3, 10 and 15 on the DTLZ4, 5, 8, and 10 on the DLTZ5, and 3, 5, and 8 on the DTLZ6. Moreover, the objective 5 on the DTLZ1, objective 15 on the DTLZ2, objectives 3 and 5 on the DTLZ4, objectives 3 and 15 on the DTLZ5, and objectives 10 and 15 on the DTLZ6 achieve the second best results. However, the performances of objective 3 on the DTLZ2 and 8 on the DTLZ3 are poor. Although NSGA-III, VAEA, RVEA, and MOEA/D can obtain optimal values for a particular dimension in the function, NSGA-III-WA has the best overall performance considering all dimensional objective results.  Table 9 shows summary of statistical test results from Table 6. As can be seen from the table, NSGA-III-WA hypervolume performance is better than the other five algorithms.

**Figure 3:**
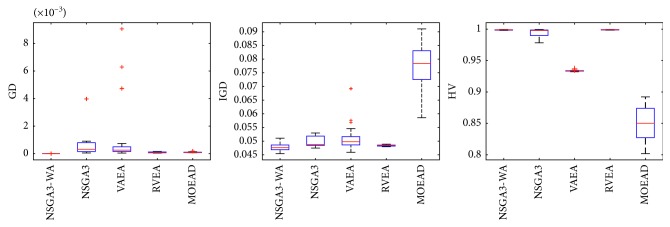
Boxplots of GD, IGD, and HV index by the four algorithms with 5 objectives on DTLZ1 problem.

**Figure 4:**
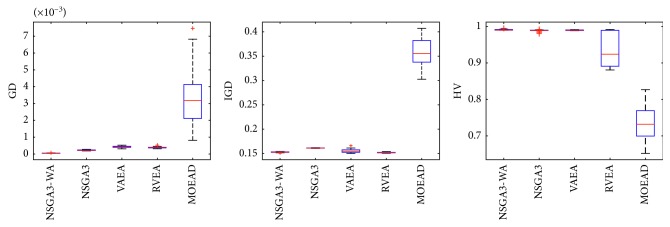
Boxplots of GD, IGD, and HV index by the four algorithms with 5 objectives on DTLZ2 problem.

**Figure 5:**
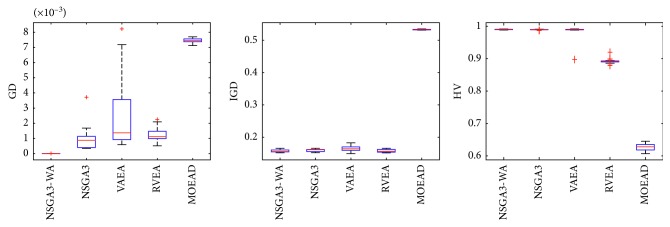
Boxplots of GD, IGD, and HV index by the four algorithms with 5 objectives on DTLZ3 problem.

**Figure 6:**
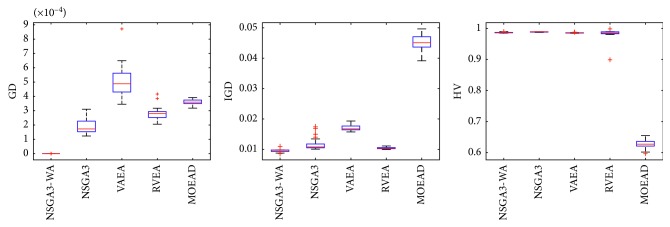
Boxplots of GD, IGD, and HV index by the four algorithms with 5 objectives on DTLZ4 problem.

**Figure 7:**
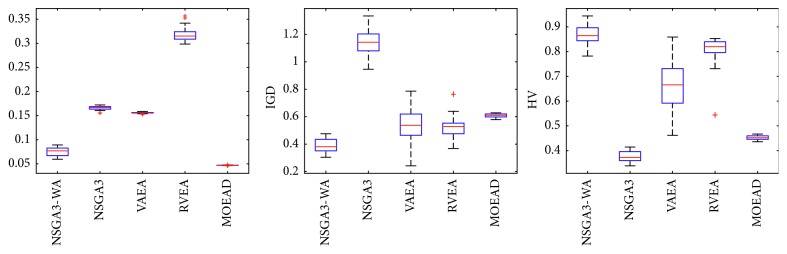
Boxplots of GD, IGD, and HV index by the four algorithms with 5 objectives on DTLZ5 problem.

**Figure 8:**
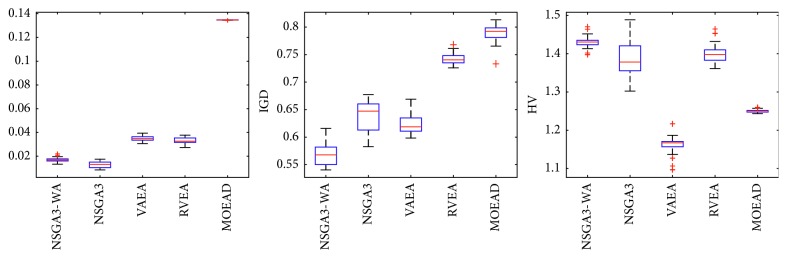
Boxplots of GD, IGD, and HV index by the four algorithms with 5 objectives on DTLZ6 problem.

**Figure 9:**
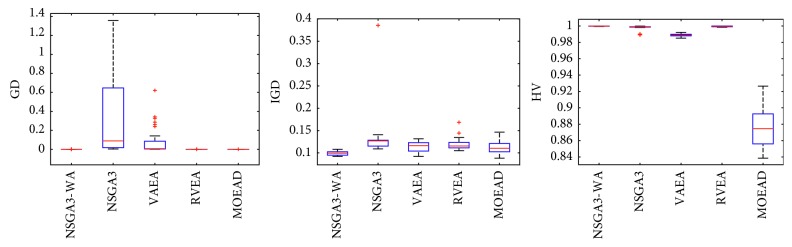
Boxplots of GD, IGD, and HV index by the four algorithms with 15 objectives on DTLZ1 problem.

**Figure 10:**
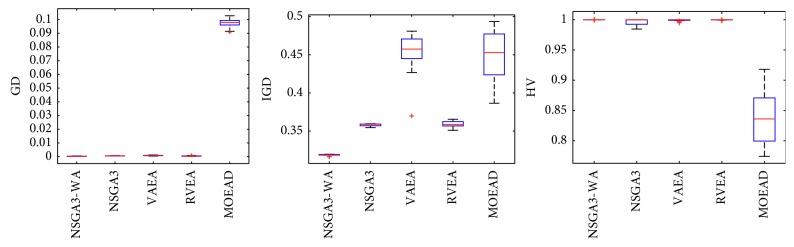
Boxplots of GD, IGD, and HV index by the four algorithms with 15 objectives on DTLZ2 problem.

**Figure 11:**
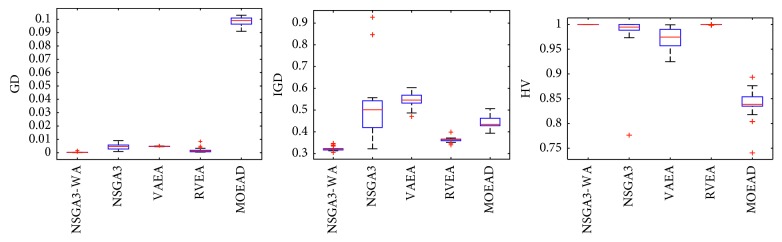
Boxplots of GD, IGD, and HV index by the four algorithms with 15 objectives on DTLZ3 problem.

**Figure 12:**
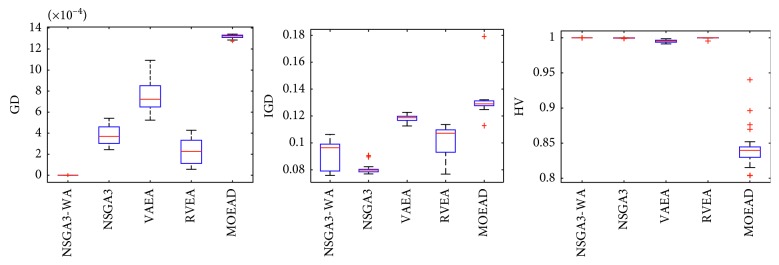
Boxplots of GD, IGD, and HV index by the four algorithms with 15 objectives on DTLZ4 problem.

**Figure 13:**
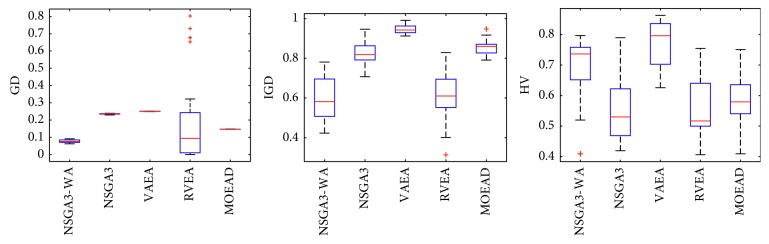
Boxplots of GD, IGD, and HV index by the four algorithms with 15 objectives on DTLZ5 problem.

**Figure 14:**
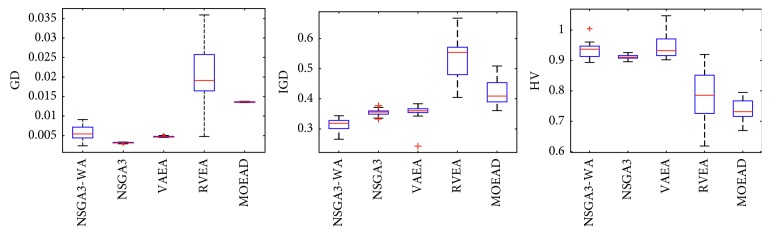
Boxplots of GD, IGD, and HV index by the four algorithms with 15 objectives on DTLZ6 problem.

In order to express the effect of the algorithm more intuitively, the performance of the algorithm is presented in the form of a box diagram. Due to space limitations, only the analysis of the box diagrams of the four algorithms under five goals and fifteen goals is given here. Figures 3–8 show the performance box diagram under the four goals, and Figures 9–14 show the performance box diagram under the fifteen goals. Each box diagram is calculated by inputting 30 independent running results. It reflects the median, maximum, minimum, upper quartile, lower quartile, and outliers of the five algorithms on indicators GD, IGD, and HV.

From Figures 3–8, it can be seen that NSGA-III-WA can achieve better results when dealing with most test problems. Its convergence and breadth are significantly better than the other five algorithms. The overall performance indicators achieve the best results on the DTLZ1 and DTLZ4 and get the second best results on the DTLZ2 and DTLZ3. Although the minimum value is obtained on the DTLZ5 and DTLZ6, there exist abnormal values, indicating that the algorithm is relatively unstable. This is because DTLZ5 and DTLZ6 test the ability of the algorithm to converge to a straight line, while NSGA-III-WA needs to build a hypersurface, so it cannot converge to a curve well. However, the overall robustness of NSGA-III-WA is relatively better with all test function results.

From Figures 9–14, it can be seen that the NSGA-III-WA has the ability to handle most problems under the 15 objectives. The convergence on the DTLZ1-DTLZ5 is significantly better than the other five algorithms. The overall performance obtains the best results on the DTLZ1-DTLZ3, DTLZ5, and DTLZ6. The NSGA-III-WA under the 15 objectives can get the minimum on DTLZ5 and DTLZ6 but is relatively unstable. The breadth achieves the best results on the DTLZ1, DTLZ3, and DTLZ4 and gets the second best results on the DTLZ2, DTLZ5, and DTLZ6, and there exist abnormal values. On the 15 objectives, it is evident that the outliers of each algorithm increase. That is explained by the fact that the stability of algorithms in the high-dimensional space will decline due to the increase of the spatial breadth. Depending on the results of all test functions, NSGA-III-WA has better stability.

**Figure 15:**
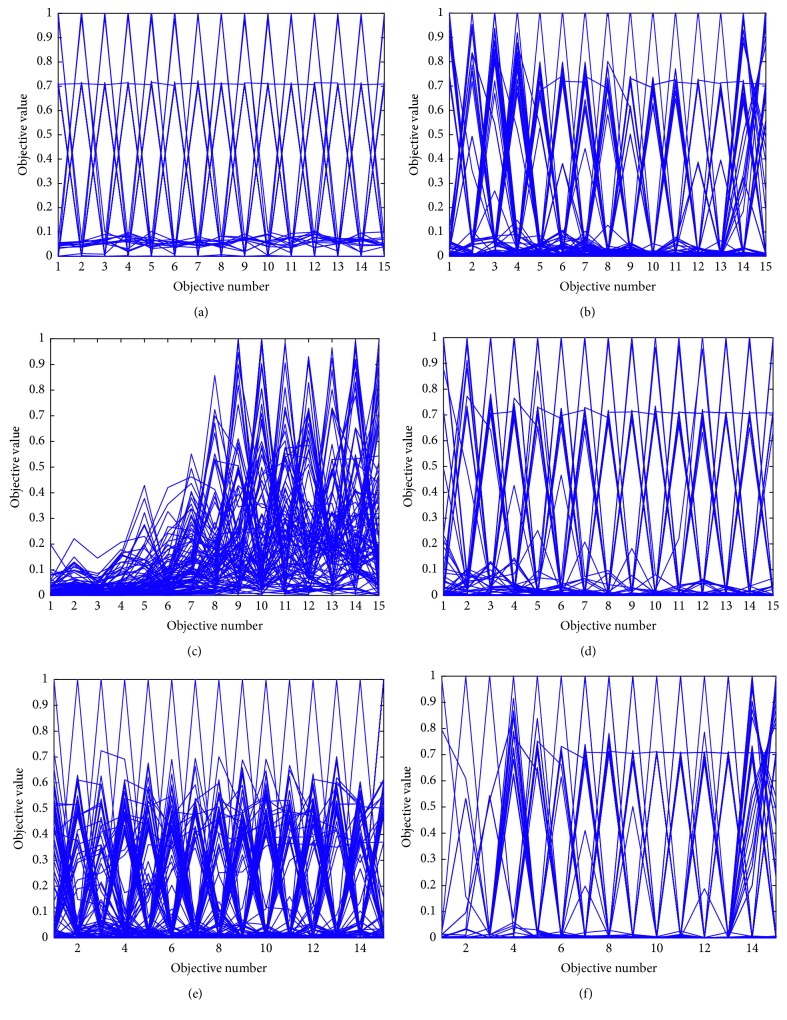
Parallel graph of the final solution set of each algorithm on the 15-objective DTLZ2 test problem. (a) NSGA-III-WA. (b) NSGA-III. (c) MOEA/D. (d) RVEA. (e) VAEA. (h) MOEA/D-M2M.

In order to visually reflect the distribution of the solution set in the high-dimensional target space, parallel coordinates are used to visualize the high-dimensional data as shown in Figure 15.

From Figure 15, it can be seen that NSGA-III-WA and RVEA find the final solution set in this problem to be similar in convergence and distribution. In contrast, MOEA/D-M2M and NSGA-III are slightly less distributed than the above three algorithms. VAEA finds that the distribution of the solution is poor. MOEA/D appears the concentrated solution. Lose extreme solutions at 12 objectives and the distribution of MOEA/D is seriously missing.

#### 5.2.2. Testing and Analysis of WFG Series Functions

The performance indicators of the WFG test function are mainly IGD and HV indicators. Therefore, this section tests the WFG1-4 test instance and analyzes the results, as shown in Tables 10–13.

From the results in Table 10, it can be seen that NSGA-III-WA can handle most of the considered examples well. In particular, it achieved the best overall performance on the objectives 3, 5, and 10 on WFG2 instances and the objectives 5, 8, and 15 on WFG3 instances. In addition, it achieves the best performance on the objective 15 on WFG8 and the objectives 3 and 8 on WFG9. The VAEA performed well on the objective 8 on WFG1 and WFG2 test instances and also achieved good results on the objectives 3 and 10 on WFG3 and the objective 4 on WFG3. RVEA obtains the best IGD value on the objective 3 on WFG1 and the objectives 5 and 10 on WFG4. It is worth noting that RVEA performs poorly for WFG2 and WFG3 instances. But it performs relatively well compared to the NSGA3 and MOEA/D algorithms. NSGA-III and MOEA/D-M2M typically have moderate performance on most WFG problems, and good results can only be achieved on specific WFG test instances. MOEA/D does not produce satisfactory results in all WFG test instances. As the number of objectives increases, the results gradually deteriorate.  Table 11 shows summary of statistical test results from Table 10. It can be seen from the table that the performance of NSGA-III-WA is significantly better than that of the other five algorithms.

From the results in Table 12, it can be seen that NSGA-III-WA has obtained the best performance for most of the high-dimensional objective problems. NSGA-III works well on the WFG1 and WFG2 test instances, and VAEA also gets good results on the objectives 10 and 15 on WFG3 test instances. RVEA obtains the best HV value on the objectives 3, 5, and 10 on WFG4. MOEA/D and MOEA/D-M2M are not quite effective on these five instances.  Table 13 shows summary of statistical test results from Table 12. The three-dimensional performance of the NSGA-III-WA algorithm is not very prominent. The performance under the eight-dimensional algorithm is the same as that of the RVEA algorithm, but the NSGA-III-WA algorithm can achieve better performance in high-dimensional objective problems. In general, the NSGA-III-WA algorithm outperforms the other five algorithms in this performance.

In summary, after comparing the test results of GD, IGD, and HV performance, the performance of the NSGA-III-WA algorithm is superior.

## 6. Conclusion

This paper proposes a many-objective optimization algorithm based on weight vector adjustment, which increases the individual's ability to evolve through new differential evolution strategies, and at the same time, dynamically adjust the weight vector by means of the *K*-means to make the weight vector as evenly distributed as possible on the objective surface. The NSGA-III-WA algorithm has good convergence ability and good distribution. To prove its effectiveness, the NSGA-III-WA is experimentally compared with the other five most advanced algorithms on the DTLZ test set and WFG test instances. The experimental results show that the proposed NSGA-III-WA performs well on the DTLZ test set and WFG test instances we studied, and the obtained solution set has good convergence and distribution. However, the proposed algorithm has high complexity and it only plays the role of alleviating sensitive frontiers. Further research will be conducted on the above problems.

## Figures and Tables

**Algorithm 1:**
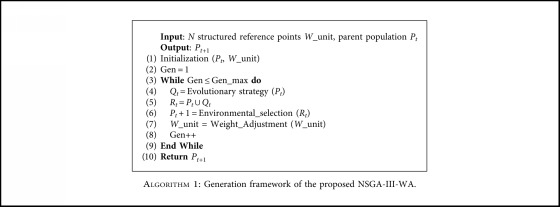
Generation framework of the proposed NSGA-III-WA.

**Algorithm 2:**
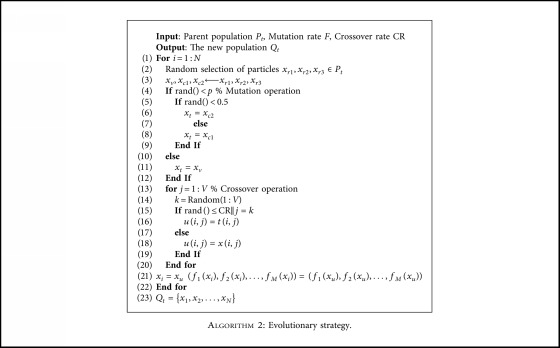
Evolutionary strategy.

**Algorithm 3:**
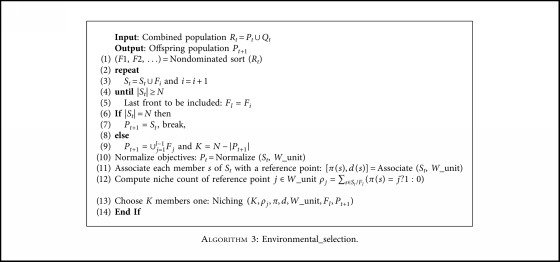
Environmental_selection.

**Algorithm 4:**
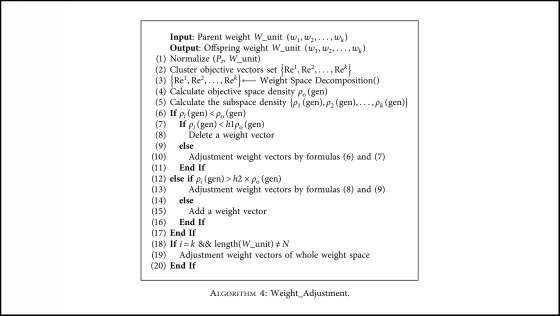
Weight_Adjustment.

**Table 1:** The population size (*N*) for different numbers of objectives.

Number of objectives *M*	Segment parameter *p*	Population size *N*
3	12	92
5	6	212
8	*p*_1_=3, *p*_2_=2	156
10	*p*_1_=2, *p*_2_=2	112
15	*p*_1_=2, *p*_2_=1	136

**Table 2:** MFE times for different numbers of objectives.

Test instance	*M*=3	*M*=5	*M*=8	*M*=10	*M*=15
DTLZ1	36,800	127,200	117,000	112,000	204,000
DTLZ2	23,000	74,200	78,000	84,000	136,000
DTLZ3	92,000	212,000	156,000	168,000	272,000
DTLZ4	55,200	212,000	195,000	224,000	408,000
DTLZ5	55,200	212,000	187,200	168,000	272,000
DTLZ6	36,800	74,200	117,000	224,000	272,000
WFG1∼4	36,800	127,200	195,000	224,000	408,000

**Table 3:** Parameter values used in NSGA-III and MOEA/D.

Parameters	NSGA-III	MOEA/D
Crossover probability *p*_c_	1	1
Variation probability *p*_m_	1/*V*	1/*V*
Cross-distribution index *η*_c_	30	20
Variance distribution index *η*_m_	20	20

**Table 4:** The GD average and standard deviation of NSGA-III-WA and other five algorithms on DTLZ1-6 testing problems.

Problem	*M*	NSGA-III-WA	NSGA-III	VAEA	RVEA	MOEA/D	MOEA/D-M2M
DTLZ1	3	**2.003*e* − 06 (1.017*e* − 05)**	9.210*e* − 04 + (2.322*e* − 04)	1.776*e* − 04 + (8.957*e* − 05)	1.091*e* − 03 + (2.154*e* − 03)	1.799*e* − 04 + (1.533*e* − 04)	6.851*e* − 03 + (8.316*e* − 03)
	5	**7.400*e* − 08 (3.413*e* − 08)**	1.543*e* − 04 + (2.730*e* − 04)	1.077*e* − 04 + (1.656*e* − 04)	4.839*e* − 04 + (3.785*e* − 05)	4.745*e* − 05 + (3.232*e* − 05)	3.885*e* − 03 + (3.274*e* − 03)
	8	**7.121*e* − 06 (4.504*e* − 06)**	2.045*e* − 03 + (1.882*e* − 03)	1.722*e* − 03 + (2.746*e* − 03)	1.225*e* − 04 + (5.246*e* − 05)	1.524*e* − 04 + (9.587*e* − 05)	7.884*e* − 03 + (6.720*e* − 03)
	10	**3.065*e* − 06 (4.042*e* − 06)**	4.618*e* − 03 + (3.393*e* − 03)	2.259*e* − 03 + (9.641*e* − 04)	2.637*e* − 03 + (1.233*e* − 04)	2.187*e* − 04 + (2.477*e* − 04)	2.199*e* − 02 + (6.495*e* − 03)
	15	**1.639*e* − 07 (2.239*e* − 07)**	7.531*e* − 02 + (8.504*e* − 03)	3.695*e* − 03 + (1.214*e* − 03)	1.920*e* − 04 + (1.682*e* − 04)	1.386*e* − 04 + (9.843*e* − 05)	4.632*e* − 02 + (1.382*e* − 03)

DTLZ2	3	**1.715*e* − 06 (6.988*e* − 07)**	1.269*e* − 04 + (1.679*e* − 05)	2.657*e* − 04 + (6.309*e* − 05)	4.557*e* − 04 + (7.907*e* − 05)	3.918*e* − 03 + (1.464*e* − 03)	2.926*e* − 04 + (3.825*e* − 05)
	5	**6.131*e* − 05 (1.146*e* − 05)**	2.524*e* − 04 + (2.555*e* − 05)	4.729*e* − 04 + (5.229*e* − 05)	3.822*e* − 04 + (4.425*e* − 05)	9.614*e* − 02 + (1.702*e* − 03)	2.736*e* − 03 + (3.057*e* − 03)
	8	**2.972*e* − 04 (3.302*e* − 05)**	6.529*e* − 04 + (8.548*e* − 05)	6.463*e* − 04 + (1.351*e* − 04)	5.385*e* − 04 + (9.497*e* − 05)	3.303*e* − 03 + (5.187*e* − 05)	1.778*e* − 03 + (3.743*e* − 04)
	10	**6.169*e* − 04 (1.346*e* − 04)**	1.139*e* − 03 + (2.172*e* − 04)	7.390*e* − 04 + (2.642*e* − 04)	9.629*e* − 04 + (1.761*e* − 04)	6.532*e* − 03 + (8.470*e* − 04)	2.556*e* − 03 + (4.978*e* − 04)
	15	**2.192*e* − 04 (1.070*e* − 04)**	5.657*e* − 04 + (9.716*e* − 05)	7.725*e* − 04 + (1.826*e* − 04)	4.202*e* − 04 + (2.084*e* − 04)	9.719*e* − 02 + (2.696*e* − 03)	1.201*e* − 02 + (3.749*e* − 04)

DTLZ3	3	**2.224*e* − 10 (2.411*e* − 06)**	5.619*e* − 04 + (1.986*e* − 04)	1.827*e* − 04 + (5.711*e* − 04)	3.788*e* − 03 + (1.353*e* − 03)	3.971*e* − 03 + (2.979*e* − 05)	1.157*e* − 03 + (6.428*e* − 04)
	5	**2.942*e* − 06 (3.373*e* − 06)**	8.990*e* − 04 + (4.012*e* − 04)	2.232*e* − 03 + (1.227*e* − 03)	1.262*e* − 03 + (2.190*e* − 04)	7.459*e* − 03 + (1.364*e* − 04)	7.998*e* − 03 + (3.265*e* − 04)
	8	**1.153*e* − 04 (3.104*e* − 05)**	4.541*e* − 03 + (3.786*e* − 04)	3.961*e* − 03 + (9.325*e* − 04)	1.901*e* − 03 + (7.506*e* − 04)	3.127*e* − 02 + (9.883*e* − 04)	3.481*e* − 02 + (2.819*e* − 03)
	10	**2.368*e* − 04 (9.563*e* − 05)**	5.926*e* − 03 + (2.259*e* − 03)	4.713*e* − 03 + (2.284*e* − 03)	9.694*e* − 03 + (2.543*e* − 03)	5.910*e* − 02 + (2.794*e* − 03)	3.009*e* − 02 + (2.295*e* − 03)
	15	**2.508*e* − 04 (2.785*e* − 04)**	4.179*e* − 03 + (2.756*e* − 03)	7.510*e* − 03 + (4.256*e* − 04)	8.655*e* − 04 + (1.074*e* − 03)	9.834*e* − 02 + (3.003*e* − 03)	5.392*e* − 02 + (2.949*e* − 03)

DTLZ4	3	**4.345*e* − 09 (4.389*e* − 09)**	4.611*e* − 04 + (2.188*e* − 04)	2.790*e* − 04 + (1.033*e* − 04)	2.852*e* − 04 + (1.749*e* − 04)	4.547*e* − 03 + (1.739*e* − 03)	1.061*e* − 04 + (3.147*e* − 05)
	5	**7.963*e* − 15 (3.060*e* − 14)**	1.905*e* − 04 + (5.277*e* − 05)	5.008*e* − 04 + (1.074*e* − 04)	2.794*e* − 04 + (4.251*e* − 05)	3.596*e* − 04 + (1.854*e* − 05)	5.092*e* − 04 + (2.427*e* − 04)
	8	**3.605*e* − 12 (1.607*e* − 11)**	4.234*e* − 04 + (7.418*e* − 05)	7.629*e* − 04 + (1.203*e* − 04)	6.762*e* − 04 + (1.645*e* − 04)	7.233*e* − 03 + (2.509*e* − 04)	3.038*e* − 03 + (3.791*e* − 04)
	10	**3.272*e* − 16 (9.275*e* − 16)**	5.561*e* − 04 + (9.294*e* − 05)	8.086*e* − 04 + (2.006*e* − 04)	1.084*e* − 03 + (2.514*e* − 04)	1.060*e* − 03 + (2.939*e* − 05)	2.981*e* − 03 + (5.370*e* − 04)
	15	**4.437*e* − 15 (2.404*e* − 14)**	3.722*e* − 04 + (8.070*e* − 05)	7.542*e* − 04 + (1.518*e* − 04)	2.257*e* − 04 + (1.222*e* − 04)	1.319*e* − 03 + (4.983*e* − 05)	3.659*e* − 03 + (2.773*e* − 04)

DTLZ5	3	1.400*e* − 01 (7.900*e* − 03)	1.344*e* − 01 + (9.360*e* − 03)	1.962*e* − 01 + (1.695*e* − 02)	2.065*e* − 01 + (1.245*e* − 02)	5.394*e* − 01 + (1.339*e* − 03)	**5.097*e* − 02 − (2.371*e* − 03)**
	5	7.633*e* − 02 (4.638*e* − 03)	1.659*e* − 01 + (1.039*e* − 03)	1.559*e* − 01 + (8.369*e* − 04)	3.419*e* − 01 + (5.779*e* − 03)	4.709*e* − 02 = (3.682*e* − 03)	**4.543*e* − 02 − (5.821*e* − 04)**
	8	**7.580*e* − 02 (6.965*e* − 03)**	1.885*e* − 01 + (4.210*e* − 03)	2.219*e* − 01 + (1.306*e* − 03)	5.373*e* − 01 + (2.058*e* − 02)	8.841*e* − 02 + (1.342*e* − 03)	8.630*e* − 02 = (3.746*e* − 03)
	10	**7.731*e* − 02 (6.006*e* − 03)**	2.259*e* − 01 + (4.640*e* − 03)	2.725*e* − 01 + (8.818*e* − 04)	3.896*e* − 01 + (3.558*e* − 03)	1.241*e* − 01 + (1.379*e* − 03)	9.947*e* − 02 + (1.385*e* − 03)
	15	**7.714*e* − 02 (7.846*e* − 03)**	2.352*e* − 01 + (2.731*e* − 03)	2.503*e* − 01 + (7.531*e* − 04)	1.196*e* − 01 + (4.752*e* − 02)	1.468*e* − 01 + (8.034*e* − 04)	9.665*e* − 02 + (4.543*e* − 03)

DTLZ6	3	**9.107*e* − 02 (1.067*e* − 02)**	1.605*e* − 01 + (1.225*e* − 02)	1.647*e* − 01 + (1.689*e* − 02)	2.299*e* − 01 + (4.912*e* − 03)	3.661*e* − 01 + (3.201*e* − 03)	2.010*e* − 01 + (3.569*e* − 03)
	5	1.714*e* − 02 (2.046*e* − 03)	**1.279*e* − 02 − (2.679*e* − 03)**	3.713*e* − 02 + (2.098*e* − 03)	3.492*e* − 02 + (2.748*e* − 03)	1.346*e* − 01 + (1.267*e* − 04)	6.235*e* − 02 + (8.714*e* − 04)
	8	1.482*e* − 02 (3.358*e* − 03)	**4.119*e* − 03 − (4.968*e* − 04)**	7.671*e* − 02 + (6.004*e* − 03)	1.448*e* − 02 = (8.183*e* − 03)	1.034*e* − 01 + (6.880*e* − 03)	2.821*e* − 02 + (3.728*e* − 03)
	10	1.868*e* − 02 (4.510*e* − 03)	**1.331*e* − 03 − (3.501*e* − 04)**	7.597*e* − 03 − (2.829*e* − 04)	1.891*e* − 02 = (8.185*e* − 03)	5.831*e* − 02 + (2.794*e* − 03)	1.495*e* − 02 − (3.746*e* − 03)
	15	5.735*e* − 03 (4.757*e* − 04)	**3.154*e* − 03 − (1.438*e* − 04)**	4.743*e* − 03 − (1.742*e* − 04)	1.701*e* − 02 + (9.023*e* − 03)	1.361*e* − 02 + (1.011*e* − 04)	3.109*e* − 02 + (7.158*e* − 02)

# +/=/−		—	26/0/4	28/0/2	28/2/0	29/1/0	26/1/3

**Table 5:** The IGD average and standard deviation of NSGA-III-WA and other five algorithms on DTLZ1-6 testing problems.

Problem	*M*	NSGA-III-WA	NSGA-III	VAEA	RVEA	MOEA/D	MOEA/D-M2M
DTLZ1	3	3.148*e* − 02 (6.732*e* − 04)	**2.096*e* − 02** = (6.245*e* − 04)	7.776*e* − 02 + (8.086*e* − 04)	6.202*e* − 02 + (2.796*e* − 03)	4.086*e* − 02 + (7.159*e* − 03)	4.315*e* − 02 + (5.569*e* − 03)
	5	**4.781*e* − 02** (1.445*e* − 04)	6.547*e* − 02 + (1.645*e* − 04)	5.203*e* − 02 + (2.858*e* − 04)	4.840*e* − 02 + (2.853*e* − 04)	7.737*e* − 02 + (3.165*e* − 04)	1.086*e* − 01 + (1.434*e* − 03)
	8	8.196*e* − 02 (3.003*e* − 03)	9.294*e* − 02 + (1.489*e* − 03)	9.351*e* − 01 + (4.178*e* − 03)	**7.720*e* − 02 − **(5.637*e* − 03)	1.149*e* − 01 + (9.790*e* − 03)	1.489*e* − 01 + (5.276*e* − 03)
	10	**9.134*e* − 02** (6.039*e* − 03)	1.309 *e* − 01 + (4.313*e* − 03)	1.119 *e* − 01 + (1.114*e* − 03)	1.142*e* − 01 + (2.156*e* − 03)	1.022*e* − 01 + (1.435*e* − 04)	2.464*e* − 01 + (3.592*e* − 03)
	15	**9.923*e* − 02** (1.501*e* − 03)	1.324*e* − 01 + (2.857*e* − 03)	1.136*e* − 01 + (4.151*e* − 03	1.188*e* − 01 + (2.306*e* − 03)	1.132*e* − 01 + (4.539*e* − 03)	1.382*e* − 01 + (1.736*e* − 03)

DTLZ2	3	5.474*e* − 02 (2.074*e* − 04)	**5.452*e* − 02** = (5.829*e* − 04)	5.637*e* − 02 + (4.368*e* − 04)	5.490*e* − 02 = (1.888*e* − 04)	6.392*e* − 02 + (7.698*e* − 04)	9.412*e* − 02 + (2.835 *e* − 03)
	5	1.527*e* − 01 (8.099*e* − 04)	1.612*e* − 01 + (3.528*e* − 04)	1.553*e* − 01 + (4.447*e* − 03)	**1.519*e* − 01 −** (8.868*e* − 04)	3.486*e* − 01 + (1.318*e* − 03)	2.095*e* − 01 + (5.578*e* − 03)
	8	**2.598*e* − 01** (3.285*e* − 03)	2.675*e* − 01 + (7.748*e* − 04)	2.979*e* − 01 + (5.934*e* − 03)	2.617*e* − 01 = (4.335*e* − 03)	3.500*e* − 01 + (2.287*e* − 03)	4.494*e* − 01 + (5.147*e* − 03)
	10	**3.286*e* − 01** (1.116*e* − 03)	3.570*e* − 01 + (9.137*e* − 03)	3.574*e* − 01 + (1.895*e* − 03)	4.600*e* − 01 + (1.013*e* − 03)	4.009*e* − 01 + (6.283*e* − 04)	4.603*e* − 01 + (5.291*e* − 03)
	15	**3.188*e* − 01** (3.626*e* − 04)	3.580*e* − 01 + (8.035*e* − 04)	4.547*e* − 01 + (2.230*e* − 03)	3.592*e* − 01 + (1.777*e* − 03)	4.596*e* − 01 + (7.071*e* − 03)	4.583*e* − 01 + (3.264*e* − 03)

DTLZ3	3	5.893*e* − 02 (7.098*e* − 04)	9.937*e* − 02 + (8.864*e* − 04)	**5.593*e* − 02 −** (1.972*e* − 03)	6.608*e* − 02 + (4.416*e* − 03)	6.385*e* − 02 + (1.490*e* − 03)	9.495*e* − 02 + (1.291*e* − 03)
	5	1.671*e* − 01 (3.529*e* − 03)	**1.598*e* − 01 −** (4.145*e* − 03)	1.650*e* − 01 + (9.129*e* − 03)	1.583*e* − 01 = (4.052*e* − 03)	5.327*e* − 01 + (1.052*e* − 03)	5.158*e* − 01 + (4.742*e* − 03)
	8	**2.857*e* − 01** (2.753*e* − 02)	4.185*e* − 01 + (1.043*e* − 01)	3.706*e* − 01 + (5.212*e* − 02)	3.117*e* − 01 + (2.923*e* − 02)	4.196*e* − 01 + (4.810*e* − 02)	4.032*e* − 01 + (8.360*e* − 02)
	10	**3.252*e* − 01** (1.778*e* − 02)	4.751*e* − 01 + (1.321*e* − 02)	6.767*e* − 01 + (4.540*e* − 02)	3.835*e* − 01 + (1.495*e* − 02)	4.401*e* − 01 + (4.446*e* − 03)	7.313*e* − 01 + (5.746*e* − 02)
	15	**3.225*e* − 01** (5.028*e* − 03)	5.076*e* − 01 + (4.409*e* − 02)	5.469*e* − 01 + (2.917*e* − 02)	3.636*e* − 01 + (7.496*e* − 03)	4.414*e* − 01 + (2.664*e* − 02)	5.743*e* − 01 + (5.692*e* − 02)

DTLZ4	3	**2.998*e* − 03** (1.402*e* − 04)	3.685*e* − 03 + (7.272*e* − 04)	5.537*e* − 02 + (1.937*e* − 01)	3.359*e* − 03 + (2.443*e* − 04)	6.434*e* − 02 + (1.009*e* − 01)	7.938*e* − 02 + (3.162*e* − 02)
	5	**9.586*e* − 03** (6.178*e* − 04)	1.173*e* − 02 + (2.162*e* − 03)	1.704*e* − 01 + (1.054*e* − 03)	1.039*e* − 02 + (3.622*e* − 04)	4.485*e* − 02 + (2.562*e* − 03)	1.419*e* − 02 + (2.946*e* − 03)
	8	**2.820*e* − 02** (1.099*e* − 03)	3.257*e* − 02 + (2.645*e* − 03)	4.432*e* − 01 + (3.122*e* − 03)	3.082*e* − 02 + (5.237*e* − 04)	2.741*e* − 01 + (2.447*e* − 03)	4.622*e* − 01 + (4.797*e* − 03)
	10	5.562*e* − 02 (3.895*e* − 03)	**5.043*e* − 02 −** (1.118*e* − 03)	7.208*e* − 01 + (3.446*e* − 03)	5.556*e* − 02 = (4.444*e* − 03)	1.411*e* − 01 + (8.020*e* − 03)	9.292*e* − 02 + (5.714*e* − 03)
	15	9.265*e* − 02 (2.780*e* − 02)	**8.040*e* − 02 −** (2.634*e* − 03)	1.183*e* − 01 + (5.087*e* − 03)	1.002*e* − 01 + (1.266*e* − 02)	1.303*e* − 01 + (9.904*e* − 03)	1.033*e* − 01 + (3.758*e* − 03)

DTLZ5	3	1.281*e* − 01 (1.585*e* − 02)	1.143*e* − 01 − (8.659*e* − 03)	1.674*e* − 01 + (5.705*e* − 02)	2.057*e* − 01 + (3.254*e* − 03)	4.196*e* − 01 + (2.332*e* − 03)	**4.329*e* − 02 − (8.859*e* − 03)**
	5	**3.854*e* − 01** (6.196*e* − 02)	1.137*e* + 00 + (1.129*e* − 01)	5.398*e* − 01 + (1.447*e* − 01)	5.198*e* − 01 + (8.029*e* − 02)	6.048*e* − 01 + (1.373*e* − 03)	4.785*e* − 01 + (4.553*e* − 02)
	8	**3.702*e* − 01** (5.821*e* − 02)	6.228*e* − 01 + (1.042*e* − 02)	7.637*e* − 01 + (3.741*e* − 02)	4.112*e* − 01 + (4.772*e* − 02)	4.647*e* − 01 + (5.300*e* − 02)	4.697*e* − 01 + (3.117*e* − 02)
	10	3.853*e* − 01 (6.602*e* − 02)	7.052*e* − 01 + (5.501*e* − 02)	6.375*e* − 01 + (6.288*e* − 02)	**3.821*e* − 01 −** (1.585*e* − 02)	7.844*e* − 01 + (2.872*e* − 02)	5.840*e* − 01 + (3.769*e* − 02)
	15	**5.905*e* − 01** (6.090*e* − 02)	8.251*e* − 01 + (2.694*e* − 02)	9.463*e* − 01 + (1.180*e* − 02)	6.022*e* − 01 = (4.108*e* − 02)	8.558*e* − 01 + (2.129*e* − 02)	6.296*e* − 01 + (5.632*e* − 02)

DTLZ6	3	**9.766*e* − 01** (2.520*e* − 02)	1.516*e* + 00 + (9.127*e* − 02)	1.656*e* + 00 + (5.092*e* − 02)	1.303*e* + 00 + (2.028*e* − 02)	1.515*e* + 00 + (7.586*e* − 03)	1.826*e* + 00 + (3.657*e* − 03)
	5	**5.673*e* − 01** (1.851*e* − 02)	6.385*e* − 01 + (3.418*e* − 02)	6.251*e* − 01 + (9.602*e* − 03)	7.416*e* − 01 + (6.407*e* − 03)	7.880*e* − 01 + (9.136*e* − 03)	9.257*e* − 01 + (5.256*e* − 03)
	8	**5.141*e* − 01** (4.012*e* − 02)	5.233*e* − 01 + (3.393*e* − 02)	5.460*e* − 01 + (1.476*e* − 02)	5.383*e* − 01 + (3.976*e* − 02)	7.703*e* − 01 + (8.253*e* − 03)	7.437*e* − 01 + (5.732*e* − 03)
	10	4.425*e* − 01 (2.195*e* − 02)	**3.994*e* − 01 −** (2.948*e* − 02)	5.030*e* − 01 + (2.205*e* − 02)	6.184*e* − 01 + (1.084*e* − 02)	7.130*e* − 01 + (4.135*e* − 03)	6.718*e* − 01 + (5.169*e* − 02)
	15	**3.147*e* − 01** (2.727*e* − 02)	3.558*e* − 01 + (1.079*e* − 02)	3.566*e* − 01 + (2.154*e* − 02)	5.502*e* − 01 + (8.639*e* − 02)	4.214*e* − 01 + (3.918*e* − 02)	6.972*e* − 01 + (4.715*e* − 02)

#+/=/−		—	23/2/5	29/0/1	22/5/3	30/0/0	29/0/1

**Table 6:** The HV average and standard deviation of NSGA-III-WA and other five algorithms on DTLZ1-6 testing problems.

Problem	*M*	NSGA-III-WA	NSGA-III	VAEA	RVEA	MOEA/D	MOEA/D-M2M
DTLZ1	3	**9.727*e* − 01** (1.679*e* − 03)	9.661*e* − 01 = (3.208*e* − 03)	6.745*e* − 01 + (8.305*e* − 03)	9.379*e* − 01 + (9.716*e* − 03)	6.232*e* − 01 + (1.434*e* − 03)	9.595*e* − 01 + (5.082*e* − 03)
	5	9.987*e* − 01 (4.645*e* − 04)	9.941*e* − 01 + (5.491*e* − 03)	9.936*e* − 01 + (8.779*e* − 04)	**9.990*e* − 01** − (3.939*e* − 04)	8.516*e* − 01 + (5.741*e* − 03)	8.409*e* − 01+(1.297*e* − 03)
	8	**9.986*e* − 01** (1.009*e* − 03)	9.910*e* − 01 + (8.001*e* − 03)	8.763*e* − 01 + (3.096*e* − 03)	9.724*e* − 01 + (3.224*e* − 03)	8.396*e* − 01 + (4.159*e* − 03)	8.682*e* − 01 + (5.529*e* − 03)
	10	**9.983*e* − 01** (5.898*e* − 04)	9.858*e* − 01 + (1.008*e* − 03)	9.082*e* − 01 + (1.463*e* − 03)	9.972*e* − 01 = (3.197*e* − 03)	8.974*e* − 01 + (3.229*e* − 03)	8.754*e* − 01 + (2.519*e* − 03)
	15	**9.998*e* − 01** (1.572*e* − 04)	9.980*e* − 01 + (7.871*e* − 04)	9.960*e* − 01 + (1.004*e* − 03)	9.995*e* − 01 + (5.718*e* − 04)	8.830*e* − 01 + (8.141*e* − 03)	7.307*e* − 01 + (2.841*e* − 03)

DTLZ2	3	9.244*e* − 01 (2.256*e* − 03)	9.250*e* − 01 = (2.214*e* − 03)	9.231*e* − 01 + (1.889*e* − 03)	**9.251*e* − 01** − (3.107*e* − 03)	7.737*e* − 01 + (1.329*e* − 03)	8.968*e* − 01 + (2.764*e* − 03)
	5	**9.909*e* − 01** (1.504*e* − 03)	9.890*e* − 01 + (5.152*e* − 04)	9.899*e* − 01 + (9.500*e* − 04)	9.379*e* − 01 + (7.638*e* − 03)	7.323*e* − 01 + (4.672*e* − 03)	9.760*e* − 01 + (2.302*e* − 03)
	8	**9.992*e* − 01** (1.119*e* − 04)	9.984*e* − 01 + (2.264*e* − 04)	9.885*e* − 01 + (1.367*e* − 03)	9.985*e* − 01 + (2.970*e* − 03)	7.386*e* − 01 + (4.697*e* − 03)	8.922*e* − 01 + (6.281*e* − 03)
	10	**9.989*e* − 01** (4.487*e* − 04)	9.942*e* − 01 + (4.741*e* − 03)	9.969*e* − 01 + (2.531*e* − 03)	9973*e* − 01 + (3.720*e* − 03)	7.020*e* − 01 + (5.068*e* − 03)	8.815*e* − 01 + (5.413*e* − 03)
	15	9.999*e* − 01 (2.973*e* − 04)	**1.000*e* + 00** = (2.199*e* − 03)	9.998*e* − 01 + (1.731*e* − 03)	9.999*e* − 01 + (1.017*e* − 03)	8.788*e* − 01 + (7.351*e* − 03)	9.082*e* − 01 + (4.352*e* − 03)

DTLZ3	3	**9.261*e* − 01** (2.199*e* − 03)	9.202*e* − 01 = (1.894*e* − 03)	9.235*e* − 01 + (1.788*e* − 03)	9.197*e* − 01 + (4.206*e* − 03)	8.482*e* − 01 + (1.881*e* − 03)	9.062*e* − 01 + (7.041*e* − 03)
	5	**9.899*e* − 01** (1.063*e* − 03)	9.892*e* − 01 = (9.066*e* − 04)	9.865*e* − 01 = (1.690*e* − 03)	8.921*e* − 01 + (2.124*e* − 03)	6.265*e* − 01 + (7.156*e* − 03)	4.653*e* − 01 + (2.740*e* − 03)
	8	9.831*e* − 01 (1.132*e* − 03)	**9.984*e* − 01** − (2.184*e* − 04)	8.619*e* − 01 + (5.486*e* − 03)	9.981*e* − 01 − (7.039*e* − 04)	7.947*e* − 01 + (4.331*e* − 03)	5.237*e* − 01 + (2.762*e* − 03)
	10	**9.975*e* − 01** (4.712*e* − 03)	9.789*e* − 01 + (5.968*e* − 03)	8.869*e* − 01 + (1.820*e* − 02)	9.916*e* − 01 + (3.154*e* − 03)	7.193*e* − 01 + (5.231*e* − 02)	3.010*e* − 01 + (7.925*e* − 02)
	15	**1.000*e* + 00** (5.351*e* − 04)	9.998*e* − 01 + (1.778*e* − 03)	9.737*e* − 01 + (4.021*e* − 03)	9.999*e* − 01 + (3.956*e* − 04)	8.390*e* − 01 + (2.721*e* − 03)	3.417*e* − 01 + (8.294*e* − 03)

DTLZ4	3	9.252*e* − 01 (2.831*e* − 03)	8.762*e* − 01 = (6.284*e* − 03)	8.950*e* − 01 + (1.073*e* − 03)	**9.261*e* − 01** = (2.322*e* − 03)	7.646*e* − 01 + (1.483*e* − 03)	9.097*e* − 01 = (6.935*e* − 03)
	5	9.867*e* − 01 (1.377*e* − 03)	**9.887*e* − 01** − (8.484*e* − 04)	9.853*e* − 01 = (9.799*e* − 04)	9.831*e* − 01 + (1.641*e* − 03)	6.276*e* − 01 + (2.529*e* − 03)	9.861*e* − 01 = (1.544*e* − 03)
	8	9.987*e* − 01 (5.391*e* − 04)	9.987*e* − 01 = (2.606*e* − 04)	9.957*e* − 01 + (4.359*e* − 03)	**9.989*e* − 01** − (3.131*e* − 04)	7.757*e* − 01 + (5.391*e* − 04)	9.943*e* − 01 + (1.414*e* − 04)
	10	**9.998*e* − 01** (1.117*e* − 04)	**9.998*e* − 01** = (9.643*e* − 04)	9.996*e* − 01 + (5.604*e* − 04)	9.995*e* − 01 + (5.008*e* − 04)	7.350*e* − 01 + (5.951*e* − 04)	9.964*e* − 01 + (2.945*e* − 03)
	15	**1.000*e* + 00** (9.863*e* − 04)	9.999*e* − 01 + (2.858*e* − 04)	9.995*e* − 01 + (1.863*e* − 03)	9.998*e* − 01 + (8.315*e* − 04)	8.456*e* − 01 + (4.485*e* − 03)	9.927*e* − 01 + (4.372*e* − 03)

DTLZ5	3	8.370*e* − 01 (1.361*e* − 03)	8.128*e* − 01 + (5.771*e* − 03)	8.049*e* − 01 + (2.157*e* − 03)	**8.689*e* − 01** − (1.159*e* − 03)	8.094*e* − 01 + (1.693*e* − 03)	7.330*e* − 01 − (2.916*e* − 02)
	5	**8.622*e* − 01** (3.332*e* − 02)	3.775*e* − 01 + (1.310*e* − 02)	6.676*e* − 01 + (7.426*e* − 02)	8.057*e* − 01 + (2.957*e* − 02)	4.525*e* − 01 + (9.054 *e* − 03)	8.099*e* − 01 + (3.771*e* − 03)
	8	**8.099*e* − 01** (4.163*e* − 02)	6.056*e* − 01 + (3.357*e* − 02)	5.373*e* − 01 + (4.556*e* − 02)	7.143*e* − 01 + (3.091*e* − 02)	7.205*e* − 01 + (6.349*e* − 02)	6.365*e* − 01 + (5.982*e* − 02)
	10	**7.885*e* − 01** (3.761*e* − 02)	7.052*e* − 01 + (5.501*e* − 02)	6.375*e* − 01 + (6.294*e* − 02)	6.399*e* − 01 + (9.123*e* − 02)	6.859*e* − 01 + (5.109*e* − 02)	3.786*e* − 01 + (2.764*e* − 02)
	15	6.892*e* − 01 (7.425*e* − 02)	5.436*e* − 01 + (9.687*e* − 02)	**7.714*e* − 01 −** (9.537*e* − 02)	5.691*e* − 01 + (9.341*e* − 02)	5.864*e* − 01 + (4.891*e* − 02)	5.144*e* − 01 + (2.467*e* − 02)

DTLZ6	3	**1.079*e* + 00** (1.335*e* − 03)	1.043*e* + 00 + (4.541*e* − 03)	1.056*e* + 00 + (4.296*e* − 03)	9.258*e* − 01 + (9.737*e* − 03)	9.493*e* − 01 + (8.997*e* − 03)	1.041*e*+00 = (3.657*e* − 03)
	5	**1.429*e* + 00** (1.647*e* − 02)	1.384*e* + 00 + (5.443*e* − 02)	1.166*e* + 00 + (2.272*e* − 02)	1.402*e* + 00 + (3.644*e* − 02)	1.248*e* + 00 + (2.181*e* − 03)	1.275*e*+00 + (4.681*e* − 03)
	8	**1.468*e* + 00** (5.101*e* − 02)	1.416*e* + 00 + (2.891*e* − 02)	1.213*e* + 00 + (8.664*e* − 02)	1.176*e* + 00 + (9.943*e* − 02)	1.095*e* + 00 + (2.064*e* − 02)	8.201*e* − 01 + (7.193*e* − 02)
	10	1.123*e* + 00 (9.184*e* − 02)	**1.127*e* + 00** = (5.759*e* − 02)	9.897*e* − 01 + (2.878*e* − 02)	9.816*e* − 01 + (1.076*e* − 02)	8.064*e* − 01 + (4.445*e* − 02)	9.322*e* − 01 + (4.926*e* − 02)
	15	9.319*e* − 01 (2.287*e* − 02)	9.107*e* − 01 + (7.325*e* − 03)	**9.461*e* − 01** = (4.023*e* − 02)	7.651*e* − 01 + (9.930*e* − 02)	7.378*e* − 01 + (3.547*e* − 02)	7.194*e* − 01 + (3.271*e* − 02)

#+/=/−		—	19/9/2	26/3/1	23/2/5	30/0/0	26/3/1

**Table 7:** Summary of statistical test results in Table 4.

NSGA-III-WA	Objective number	vs. NSGA-III	vs. VAEA	vs. RVEA	vs. MOEA/D	vs. MOEA/D-M2M
GD	3	+: 6, =: 0, −: 0	+: 6, =: 0, −: 0	+: 6, =: 0, −: 0	+: 6, =: 0, −: 0	+: 5, =: 0, −: 1
	5	+: 5, =: 0, −: 1	+: 6, =: 0, −: 0	+: 6, =: 0, −: 0	+: 5, =: 1, −: 0	+: 5, =: 0, −: 1
	8	+: 5, =: 0, −: 1	+: 6, =: 0, −: 0	+: 5, =: 1, −: 0	+: 6, =: 0, −: 0	+: 5, =: 1, −: 0
	10	+: 5, =: 0, −: 1	+: 5, =: 0, −: 1	+: 5, =: 1, −: 0	+: 6, =: 0, −: 0	+: 5, =: 0, −: 1
	15	+: 5, =: 0, −: 1	+: 5, =: 0, −: 1	+: 6, =: 0, −: 0	+: 6, =: 0, −: 0	+: 6, =: 0, −: 0

Note: “+,” “=,” and “−” represent wins, equal to, and lose.

**Table 8:** Summary of statistical test results in Table 5.

NSGA-III-WA	Objective number	vs. NSGA-III	vs. VAEA	vs. RVEA	vs. MOEA/D	vs. MOEA/D-M2M
IGD	3	+: 3, =: 2, −: 1	+: 5, =: 0, −: 1	+: 5, =: 1, −: 0	+: 6, =: 0, −: 0	+: 5, =: 0, −: 1
	5	+: 5, =: 0, −: 1	+: 6, =: 0, −: 0	+: 4, =: 1, −: 1	+: 6, =: 0, −: 0	+: 6, =: 0, −: 0
	8	+: 6, =: 0, −: 0	+: 6, =: 0, −: 0	+: 4, =: 1, −: 1	+: 6, =: 0, −: 0	+: 6, =: 0, −: 0
	10	+: 4, =: 0, −: 2	+: 6, =: 0, −: 0	+: 4, =: 1, −: 1	+: 6, =: 0, −: 0	+: 6, =: 0, −: 0
	15	+: 5, =: 0, −: 1	+: 6, =: 0, −: 0	+: 5, =: 1, −: 0	+: 6, =: 0, −: 0	+: 6, =: 0, −: 0

Note: “+,” “=,” and “−” represent wins, equal to, and lose.

**Table 9:** Summary of statistical test results in Table 6.

NSGA-III-WA	Objective number	vs. NSGA-III	vs. VAEA	vs. RVEA	vs. MOEA/D	vs. MOEA/D-M2M
HV	3	+: 2, =: 4, −: 0	+: 6, =: 0, −: 0	+: 3, =: 1, −: 2	+: 6, =: 0, −: 0	+: 3, =: 2, −: 1
	5	+: 4, =: 1, −: 1	+: 4, =: 2, −: 0	+: 5, =: 1, −: 0	+: 6, =: 0, −: 0	+: 5, =: 1, −: 0
	8	+: 5, =: 1, −: 1	+: 6, =: 0, −: 0	+: 4, =: 0, −: 2	+: 6, =: 0, −: 0	+: 6, =: 0, −: 0
	10	+: 4, =: 2, −: 0	+: 6, =: 0, −: 0	+: 5, =: 0, −: 1	+: 6, =: 0, −: 0	+: 6, =: 0, −: 0
	15	+: 5, =: 1, −: 0	+: 4, =: 1, −: 1	+: 6, =: 0, −: 0	+: 6, =: 0, −: 0	+: 6, =: 0, −: 0

Note: “+,” “=,” and “−” represent wins, equal to, and lose.

**Table 10:** The IGD average and standard deviation of NSGA-III-WA and other five algorithms on WFG1-4 testing problems.

Problem	*M*	NSGA-III-WA	NSGA-III	VAEA	RVEA	MOEA/D	MOEA/D-M2M
WFG1	3	1.171*e* + 00 (2.727*e* − 01)	1.370*e* + 00 + (3.356*e* − 01)	1.324*e* + 00 + (2.315*e* − 01)	**1.047*e* + 00 −** (2.417*e* − 01)	1.216*e* + 00 + (2.173*e* − 01)	1.211*e* + 00 + (3.725*e* − 01)
	5	2.828*e* + 00 (1.057*e* − 01)	2.927*e* + 00 + (3.726*e* − 01)	3.203*e* + 00 + (2.941*e* − 01)	3.171*e* + 00 + (3.173*e* − 01)	3.701*e* + 00 + (2.053*e* − 01)	**1.953*e* + 00** − (3.278*e* − 01)
	8	5.721*e* + 00 (1.714*e* − 01)	5.230*e* + 00 − (1.572*e* − 01)	**5.139*e* + 00 −** (1.437*e* − 01)	5.520*e* + 00 − (1.635*e* − 01)	6.623*e* + 00 + (1.052*e* − 01)	5.769*e* + 00 = (1.678*e* − 01)
	10	7.146*e* + 00 (3.182*e* − 02)	**7.071*e* + 00 −** (5.709*e* − 02)	7.238*e* + 00 + (4.083*e* − 02)	7.182*e* + 00 + (4.281*e* − 02)	9.541*e* + 00 + (3.219*e* − 01)	7.816*e* + 00 + (9.023*e* − 02)
	15	**8.942*e* + 00** (1.284*e* − 01)	9.079*e* + 00 + (1.673*e* − 01)	9.057*e* + 00 + (3.073*e* − 01)	9.149*e* + 00 + (3.726*e* − 01)	1.183*e* + 01 + (2.961*e* − 01)	1.235*e* + 00 + (3.618*e* − 01)

WFG2	3	**2.149*e* − 01** (6.137*e* − 02)	2.839*e* − 01 + (1.040*e* − 01)	3.218*e* − 01 + (8.931*e* − 02)	3.157*e* − 01 + (4.366*e* − 02)	1.317*e* + 00 + (7.013*e* − 02)	3.714*e* − 01 + (4.827*e* − 02)
	5	**5.237*e* − 01** (9.814*e* − 02)	6.125*e* − 01 + (1.375*e* − 01)	9.052*e* − 01 + (2.781*e* − 01)	7.026*e* − 01 + (1.437*e* − 01)	3.971*e* + 00 + (2.739*e* − 01)	1.411*e* + 00 + (3.894*e* − 01)
	8	2.316*e* + 00 (1.835*e* − 01)	3.146*e* + 00 + (1.379*e* − 01)	**2.007*e* + 00 −** (2.371*e* − 01)	2.572*e* + 00 + (1.638*e* − 01)	8.837*e* + 00 + (5.462*e* − 01)	2.885*e* + 00 + (4.732*e* − 01)
	10	**2.037*e* + 00** (2.171*e* − 01)	2.923*e* + 00 + (4.518*e* − 01)	3.592*e* + 00 + (4.178*e* − 01)	2.964*e* + 00 + (3.926*e* − 01)	1.027*e* + 01 + (1.001*e* + 00)	2.1416*e* + 00 = (9.287*e* − 01)
	15	5.187*e* + 00 (1.373*e* − 01)	6.223*e* + 00 + (2.381*e* − 01)	5.250*e* + 00 = (5.936*e* − 01)	4.945*e* + 00 = (3.826*e* − 01)	1.346*e* + 01 + (2.964*e* − 01)	**4.014*e* + 00** − (5.382*e* − 01)

WFG3	3	2.163*e* − 01 (2.647*e* − 02)	3.791*e* − 01 + (8.167*e* − 02)	**1.489*e* − 01 −** (6.916*e* − 03)	1.977*e* − 01 − (3.283*e* − 02)	1.793*e* − 01 − (3.034*e* − 02)	2.361*e* − 01 + (4.624*e* − 02)
	5	**4.746*e* − 01** (6.437*e* − 03)	5.274*e* − 01 + (7.136*e* − 03)	4.793*e* − 01 + (6.374*e* − 03)	4.827*e* − 01 + (5.378*e* − 03)	5.418*e* − 01 + (2.542*e* − 02)	7.633*e* − 01 + (1.091*e* − 01)
	8	**1.308*e* + 00** (2.748*e* − 02)	1.709*e* + 00 + (1.061*e* − 01)	1.427*e* + 00 + (2.135*e* − 02)	1.604*e* + 00 + (3.375*e* − 02)	1.829*e* + 00 + (4.873*e* − 02)	2.487*e* + 00 + (3.823*e* − 02)
	10	1.864*e* + 00 (2.073*e* − 02)	2.176*e* + 00 + (2.874*e* − 01)	**1.725*e* + 00 −** (3.271*e* − 02)	1.845*e* + 00 = (4.273*e* − 02)	2.966*e* + 00 + (7.627*e* − 02)	3.369*e* + 00 + (6.379*e* − 02)
	15	**2.815*e* + 00** (2.733*e* − 01)	4.206*e* + 00 + (1.537*e* − 01)	2.963*e* + 00 + (2.736*e* − 01)	3.028*e* + 00 + (1.893*e* − 01)	5.265*e* + 00 + (8.733*e* − 02)	6.738*e* + 00 + (1.284*e* − 01)

WFG4	3	**2.043*e* − 01** (2.274*e* − 03)	2.147*e* − 01 + (3.859*e* − 04)	2.317*e* − 01 + (7.352*e* − 03)	2.272*e* − 01 + (3.72*e* − 03)	2.475*e* − 01 + (3.758*e* − 03)	3.581*e* − 01 + (3.146*e* − 03)
	5	9.635*e* − 01 (3.762*e* − 03)	9.865*e* − 01 + (4.873*e* − 03)	9.535*e* − 01 + (5.378*e* − 03)	**9.526*e* − 01 −** (3.288*e* − 03)	1.284*e* + 00 + (3.725*e* − 02)	1.676*e* + 00 + (1.003*e* − 02)
	8	**3.021*e* + 00** (4.887*e* − 03)	3.262*e* + 00 + (6.256*e* − 03)	3.023*e* + 00 = (1.567*e* − 02)	3.114*e* + 00 + (6.331*e* − 03)	6.642*e* + 00 + (1.526*e* − 02)	4.6209*e* + 00 + (2.462*e* − 02)
	10	4.063*e* + 00 (1.879*e* − 02)	4.621*e* + 00 + (2.834*e* − 02)	3.982*e* + 00 = (3.274*e* − 02)	**3.870*e* + 00 −** (2.716*e* − 01)	9.826*e* + 00 + (1.873*e* − 01)	6.698*e* + 00 + (2.706*e* − 01)
	15	8.926*e* + 00 (2.768*e* − 01)	9.732*e* + 00 + (3.381*e* − 02)	**8.541*e* + 00 −** (2.834*e* − 01)	8.737*e* + 00 − (2.762*e* − 01)	1.496*e* + 01 + (9.276*e* − 03)	1.103*e* + 01 + (1.241*e* − 01)

# +/=/−		—	18/0/2	12/3/5	12/2/6	19/0/1	16/2/2

**Table 11:** Summary of statistical test results in Table 10.

NSGA-III-WA	Objective number	vs. NSGA-III	vs. VAEA	vs. RVEA	vs. MOEA/D	vs. MOEA/D-M2M
IGD	3	+: 4, =: 0, −: 0	+: 3, =: 0, −: 1	+: 2, =: 0, −: 2	+: 3, =: 0, −: 1	+: 4, =: 0, −: 0
	5	+: 4, =: 0, −: 0	+: 4, =: 0, −: 0	+: 3, =: 0, −: 1	+: 4, =: 0, −: 0	+: 3, =: 0, −: 1
	8	+: 3, =: 0, −: 1	+: 1, =: 1, −: 2	+: 3, =: 0, −: 1	+: 4, =: 0, −: 0	+: 3, =: 1, −: 0
	10	+: 3, =: 0, −: 1	+: 2, =: 1, −: 1	+: 2, =: 1, −: 1	+: 4, =: 0, −: 0	+: 3, =: 1, −: 0
	15	+: 4, =: 0, −: 0	+: 2, =: 1, −: 1	+: 2, =: 1, −: 1	+: 4, =: 0, −: 0	+: 3, =: 0, −: 1

Note: “+,” “=,” and “−” represent wins, equal to, and lose.

**Table 12:** The HV average and standard deviation of NSGA-III-WA and other five algorithms on WFG1-4 testing problems.

Problem	*M*	NSGA-III-WA	NSGA-III	VAEA	RVEA	MOEA/D	MOEA/D-M2M
WFG1	3	5.114*e* − 01 (2.425*e* − 02)	5.013*e* − 01 + (2.279*e* − 02)	**5.217*e* − 01 −** (2.725*e* − 02)	4.963*e* − 01 + (2.673*e* − 02)	4.927*e* − 01 + (9.245*e* − 03)	4.824*e* − 01 + (5.245*e* − 02)
	5	4.725*e* − 01 (4.269*e* − 03)	4.632*e* − 01 + (5.352*e* − 03)	5.172*e* − 01 − (4.281*e* − 03)	4.824*e* − 01 = (3.729*e* − 03)	**5.793*e* − 01** − (4.736*e* − 03)	4.875*e* − 01 = (4.237*e* − 03)
	8	**4.481*e* − 01** (2.724*e* − 03)	4.116*e* − 01 + (3.861*e* − 03)	4.480*e* − 01 = (3.783*e* − 03)	4.376*e* − 01 = (2.751*e* − 02)	4.472*e* − 01 = (2.747*e* − 02)	4.324*e* − 01 = (3.783*e* − 02)
	10	6.063*e* − 01 (3.217*e* − 02)	5.937*e* − 01 = (6.273*e* − 02)	5.997*e* − 01 = (4.238*e* − 02)	**6.218*e* − 01 −** (3.628*e* − 02)	4.926*e* − 01 + (1.026*e* − 01)	5.382*e* − 01 + (7.375*e* − 02)
	15	**6.279*e* − 01** (2.781*e* − 02)	6.163*e* − 01 + (3.273*e* − 02)	6.181*e* − 01 + (2.672*e* − 02)	6.197*e* − 01 + (2.418*e* − 02)	3.472*e* − 01 + (2.163*e* − 01)	4.781*e* − 01 + (1.784*e* − 01)

WFG2	3	8.373*e* − 01 (4.263*e* − 02)	**8.524*e* − 01 −** (3.279*e* − 02)	8.393*e* − 01 = (3.789*e* − 02)	8.334*e* − 01 = (2.274*e* − 02)	7.251*e* − 01 + (3.194*e* − 02)	8.425*e* − 01 − (2.785*e* − 02)
	5	**9.602*e* − 01** (3.793*e* − 02)	9.547*e* − 01 + (3.926*e* − 02)	9.482*e* − 01 + (4.821*e* − 02)	9.376*e* − 01 + (4.245*e* − 02)	9.172*e* − 01 + (8.278*e* − 02)	9.318*e* − 01 + (6.351*e* − 02)
	8	9.223*e* − 01 (2.891*e* − 02)	9.502*e* − 01 − (3.268*e* − 02)	9.172*e* − 01 + (2.753*e* − 02)	**9.514*e* − 01 −** (2.724*e* − 02)	8.702*e* − 01 + (6.932*e* − 02)	8.945*e* − 01 + (4.837*e* − 02)
	10	**9.492*e* − 01** (2.893*e* − 02)	9.471*e* − 01 + (2.062*e* − 02)	9.261*e* − 01 + (3.625*e* − 02)	9.372*e* − 01 + (1.341*e* − 01)	8.981*e* − 01 + (1.826*e* − 01)	9.148*e* − 01 + (4.782*e* − 01)
	15	**9.715*e* − 01** (2.715*e* − 02)	9.678*e* − 01 + (2.361*e* − 02)	9.483*e* − 01 + (2.936*e* − 02)	9.572*e* − 01 + (3.798*e* − 02)	7.815*e* − 01 + (3.781*e* − 01)	8.147*e* − 01 + (3.461*e* − 01)

WFG3	3	8.068*e* − 01 (3.278*e* − 03)	**8.136*e* − 01 −** (2.267*e* − 03)	7.978*e* − 01 + (4.624*e* − 03)	5.749*e* − 01 + (2.411*e* − 02)	7.371*e* − 01 + (4.267*e* − 02)	5.361*e* − 01 + (3.283*e* − 02)
	5	8.723*e* − 01 (2.798*e* − 03)	**8.825*e* − 01 −** (3.142*e* − 03)	8.734*e* − 01 = (3.682*e* − 03)	5.921*e* − 01 + (5.274*e* − 02)	7.702*e* − 01 + (9.257*e* − 02)	5.032*e* − 01 + (3.863*e* − 02)
	8	**9.267*e* − 01** (3.278*e* − 03)	9.241*e* − 01 + (5.261*e* − 03)	9.257*e* − 01 + (4.258*e* − 02)	7.026*e* − 01 + (2.794*e* − 02)	7.315*e* − 01 + (8.903*e* − 02)	8.461*e* − 01 + (6.352*e* − 02)
	10	9.349*e* − 01 (1.392*e* − 03)	9.352*e* − 01 = (3.267*e* − 03)	**9.392*e* − 01 −** (2.674*e* − 03)	5.252*e* − 01 + (2.493*e* − 02)	4.315*e* − 01 + (1.367*e* − 01)	7.947*e* − 01 + (6.375*e* − 01)
	15	9.318*e* − 01 (2.717*e* − 03)	9.264*e* − 01 + (3.257*e* − 03)	**9.381*e* − 01 −** (2.916*e* − 03)	6.735*e* − 01 + (4.784*e* − 02)	7.106*e* − 01 + (8.628*e* − 02)	5.375*e* − 01 + (7.783*e* − 02)

WFG4	3	6.997*e* − 01 (3.278*e* − 03)	6.805*e* − 01 + (2.581*e* − 03)	6.885*e* − 01 = (3.528*e* − 03)	**7.293*e* − 01 −** (3.271*e* − 03)	6.697*e* − 01 + (3.275*e* − 02)	6.019*e* − 01 + (4.251*e* − 02)
	5	8.674*e* − 01 (2.678*e* − 03)	8.640*e* − 01 = (4.216*e* − 03)	8.601*e* − 01 + (3.728*e* − 03)	**8.756*e* − 01 −** (3.782*e* − 02)	8.602*e* − 01 = (5.272*e* − 03)	8.327*e* − 01 + (6.429*e* − 03)
	8	**9.147*e* − 01** (2.791*e* − 03)	9.020*e* − 01 + (3.736*e* − 03)	9.103*e* − 01 = (3.827*e* − 03)	9.128*e* − 01 = (3.782*e* − 03)	7.502*e* − 01 + (2.861*e* − 02)	8.462*e* − 01 + (4.571*e* − 02)
	10	8.573*e* − 01 (3.728*e* − 03)	8.517*e* − 01 + (4.286*e* − 03)	8.237*e* − 01 + (3.183*e* − 03)	**8.603*e* − 01** = (4.247*e* − 03)	7.136*e* − 01 + (2.271*e* − 02)	8.354*e* − 01 + (1.180*e* − 02)
	15	**9.114*e* − 01** (1.273*e* − 03)	9.077*e* − 01 + (7.263*e* − 03)	9.105*e* − 01 = (3.726*e* − 03)	8.982*e* − 01 + (4.251*e* − 03)	4.525*e* − 01 + (1.597*e* − 01)	7.239*e* − 01 + (2.743*e* − 01)

# +/=/−		—	13/3/4	9/7/4	11/5/4	17/2/1	17/2/1

**Table 13:** Summary of statistical test results in Table 12.

NSGA-III-WA	Objective number	vs. NSGA-III	vs. VAEA	vs. RVEA	vs. MOEA/D	vs. MOEA/D-M2M
HV	3	+: 2, =: 0, −: 2	+: 1, =: 2, −: 1	+: 2, =: 1, −: 1	+: 4, =: 0, −: 0	+: 3, =: 0, −: 1
	5	+: 2, =: 1, −: 1	+: 2, =: 1, −: 1	+: 2, =: 1, −: 1	+: 2, =: 1, −: 1	+: 3, =: 1, −: 0
	8	+: 3, =: 0, −: 1	+: 2, =: 2, −: 0	+: 1, =: 2, −: 1	+: 3, =: 1, −: 0	+: 3, =: 1, −: 0
	10	+: 2, =: 2, −: 0	+: 2, =: 1, −: 1	+: 2, =: 1, −: 1	+: 4, =: 0, −: 0	+: 4, =: 0, −: 0
	15	+: 4, =: 0, −: 0	+: 2, =: 1, −: 1	+: 4, =: 0, −: 0	+: 4, =: 0, −: 0	+: 4, =: 0, −: 0

Note: “+,” “=,” and “−” represent wins, equal to, and lose.

## Data Availability

The data used to support the findings of this study are available from the corresponding author upon request.
